# Structure of the human ClC-1 chloride channel

**DOI:** 10.1371/journal.pbio.3000218

**Published:** 2019-04-25

**Authors:** Kaituo Wang, Sarah Spruce Preisler, Liying Zhang, Yanxiang Cui, Julie Winkel Missel, Christina Grønberg, Kamil Gotfryd, Erik Lindahl, Magnus Andersson, Kirstine Calloe, Pascal F. Egea, Dan Arne Klaerke, Michael Pusch, Per Amstrup Pedersen, Z. Hong Zhou, Pontus Gourdon

**Affiliations:** 1 Department of Biomedical Sciences, University of Copenhagen, Copenhagen, Denmark; 2 Department of Microbiology, Immunology & Molecular Genetics, University of California at Los Angeles, Los Angeles, California; 3 California NanoSystems Institute, University of California at Los Angeles, Los Angeles, California; 4 Department of Biology, University of Copenhagen, Copenhagen, Denmark; 5 Department of Biochemistry & Biophysics, Stockholm University, Stockholm, Sweden; 6 Department of Chemistry, Umeå University, Umeå, Sweden; 7 Department of Veterinary and Animal Sciences, University of Copenhagen, Frederiksberg, Denmark; 8 Department of Biological Chemistry, University of California at Los Angeles, Los Angeles, California; 9 Institute of Biophysics, Consiglio Nazionale delle Ricerche, Genova, Italy; 10 Department of Experimental Medical Science, Lund University, Lund, Sweden; Georgia Institute of Technology, UNITED STATES

## Abstract

ClC-1 protein channels facilitate rapid passage of chloride ions across cellular membranes, thereby orchestrating skeletal muscle excitability. Malfunction of ClC-1 is associated with myotonia congenita, a disease impairing muscle relaxation. Here, we present the cryo-electron microscopy (cryo-EM) structure of human ClC-1, uncovering an architecture reminiscent of that of bovine ClC-K and CLC transporters. The chloride conducting pathway exhibits distinct features, including a central glutamate residue (“fast gate”) known to confer voltage-dependence (a mechanistic feature not present in ClC-K), linked to a somewhat rearranged central tyrosine and a narrower aperture of the pore toward the extracellular vestibule. These characteristics agree with the lower chloride flux of ClC-1 compared with ClC-K and enable us to propose a model for chloride passage in voltage-dependent CLC channels. Comparison of structures derived from protein studied in different experimental conditions supports the notion that pH and adenine nucleotides regulate ClC-1 through interactions between the so-called cystathionine-β-synthase (CBS) domains and the intracellular vestibule (“slow gating”). The structure also provides a framework for analysis of mutations causing myotonia congenita and reveals a striking correlation between mutated residues and the phenotypic effect on voltage gating, opening avenues for rational design of therapies against ClC-1–related diseases.

## Abstract 2

Cryo-EM structures of the human ClC-1 chloride channel in different conditions reveal the overall architecture and the chloride transport pathway; the structures hint at a mechanism underlying the elusive so-called “slow-gating” regulation, and provide a framework for downstream basic and translational applications.

## Author summary

Chloride transporting CLC proteins are expressed in a wide range of organisms, and the family encompasses several members with numerous roles in human health and disease by allowing movement of chloride ions across the membranes that encapsulate cells and cellular organelles. Structurally, CLCs form dimers possessing a separate ion translocation pathway in each monomer, and they can operate as either channels or transporters that exchange chloride for protons. The CLC channel ClC-1 is critical to skeletal muscle excitability and has been proposed as a target to alleviate neuromuscular disorders. Here, we have analyzed the structure of human ClC-1 and revealed the high similarity of its ion conducting pathway to those observed in other CLC members, including prokaryotic and algal transporters. Our data suggest how ClC-1 is regulated by environmental cues to allow opening and closure, thereby permitting attenuation of muscle function. Our results help with understanding the principal determinants that govern CLC proteins and may guide downstream translational applications to combat muscle pathologies.

## Introduction

CLC proteins comprise a large family of chloride (Cl^−^)-transporting integral membrane proteins with diverse physiological functions [1–3]. The first identified human member, ClC-1, is essential for maintaining the permeability of Cl^−^ across the plasma membrane of skeletal muscle fibers, g_Cl_, accounting for approximately 80% of the resting membrane conductance and assuring precise neuronal control of muscle contraction [3]. Mutations of the ClC-1 gene cause myotonia congenita, a disease that allows a single nerve action potential to trigger a series of muscle action potentials (myotonic runs), leading to prolonged muscle contraction [4–7].

Despite distinct roles as passively conducting Cl^−^ channels and stoichiometrically coupled secondary active Cl^−^/H^+^ antiporters [2, 3], members of the CLC family share a common homodimeric core architecture, with each subunit harboring an independent ion translocation pathway [8, 9]. The molecular mechanisms of ion transport in CLC antiporters have been extensively studied functionally and structurally [8, 10–15]. Yet it is poorly understood how the antiporters and channels establish their separate functions. In addition, the complex gating processes that regulate CLC channel activity remain elusive, with only a single available structure of a channel member, namely, that of bovine ClC-K [9]. Each CLC monomer has a gate that operates independently from the other (also known as “protopore” or “fast gate”), structurally attributed to a specific glutamate, “Glu_GATE_” [10]. A slower gate controls both conducting pathways simultaneously (“common” or “slow gate”) [16], but the principles and determinants of this regulation are enigmatic. Furthermore, activity of ClC-1 is modulated by cellular cues such as phosphorylation [17], pH, and nucleotides [18, 19] in an unknown manner. Such regulation is, however, physiologically essential because intense muscle exercise leads to acidosis, resulting in an increased nucleotide sensitivity of ClC-1 and consequent reduction of g_Cl_, thereby assisting in preventing muscle fatigue [20, 21].

The recent ClC-K structure provided the first insights into the differences between CLC channels and transporters; in particular, it revealed a pore widening on the intracellular side. Yet there are surprisingly few known structural differences between the CLC channels and transporters. However, ClC-K channels exhibit only limited gating as Glu_GATE_ is missing [2, 3], and their activity has not been reported to depend on nucleotide binding [22]. Therefore key questions concerning CLC channel function and regulation remain unanswered. Furthermore, a deeper understanding on structure–phenotype relationships of myotonia-causing mutations in ClC-1 is required to shed further light on how the muscle disease is manifested at a molecular level.

## Results

**Fig 1:**
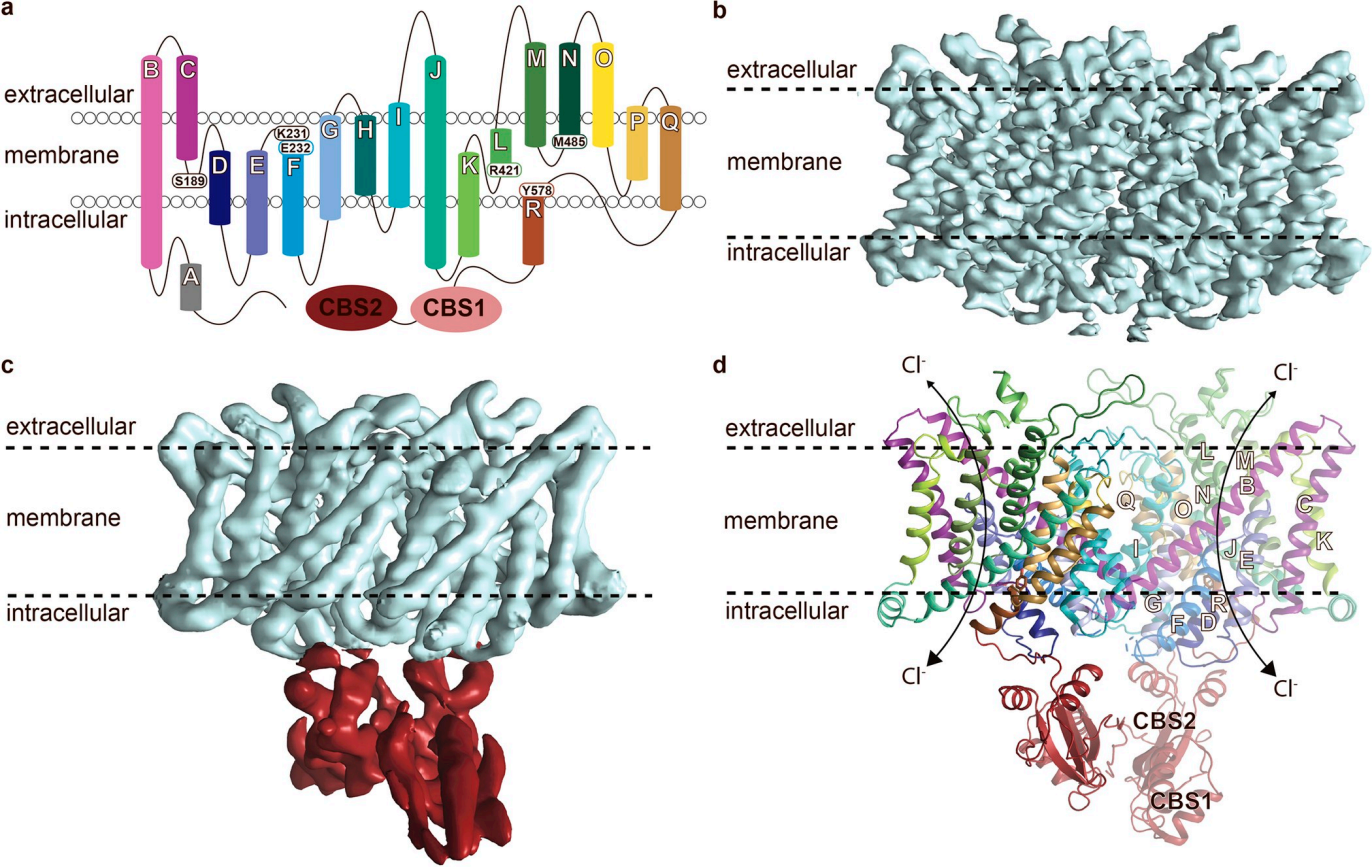
Structure of human ClC-1. (a) Overall topology of ClC-1 with 17 helices (αB to αR), 2 CBS domains, and key residues pinpointed. A single monomer is displayed for clarity. Helices are labelled with white letters throughout. (b) The 3.6 Å cryo-EM map (Map 0) from pH 7.5 covering the membrane domain only (contoured at σ = 13 in Pymol). Helix A (αA) and parts of the CBS domains were not resolved in the cryo-EM density maps. (c) Alternative cryo-EM map from pH 7.5 (Map 1) with the membrane and cytoplasmic CBS domains colored in cyan and red, respectively, shown at different contour levels (σ = 15 and 22 in Pymol, respectively). The map is filtered to 5 Å, representing the local resolution of the cytoplasmic domain (see also S2–S4 and S7 Figs). (d) Overall structure (generated using Map 1) with one of the monomers in pale colors. CBS, cystathionine-β-synthase; CLC, chloride channel; cryo-EM, cryo-electron microscopy.

Here, we have determined structures of full-length human ClC-1 using single-particle cryo-electron microscopy (cryo-EM), exploiting a purified protein sample that displays Cl^−^-dependent single-channel–derived ion conductance (S1 Fig and S1 Data). For structural characterization, sample in the presence of 100 mM Cl^−^ at pH 7.5 and in the absence of nucleotides or antibodies was initially employed (Fig 1). Three-dimensional (3D) classification of particles resulted in several different groups, of which one yielded a 3.6 Å overall resolution density map for the transmembrane domain, allowing confident model building (S2–S4 Figs). The final model represents the membrane-spanning portion (note that the N terminus and intracellular αA helix are lacking) as well as parts of two C terminal’s so-called cystathionine-β-synthase (CBS) domains present per monomer (for which some cryo-EM density is left unmodeled) and includes several features that were not observed in the ClC-K structure (S5 Fig).

**Fig 2:**
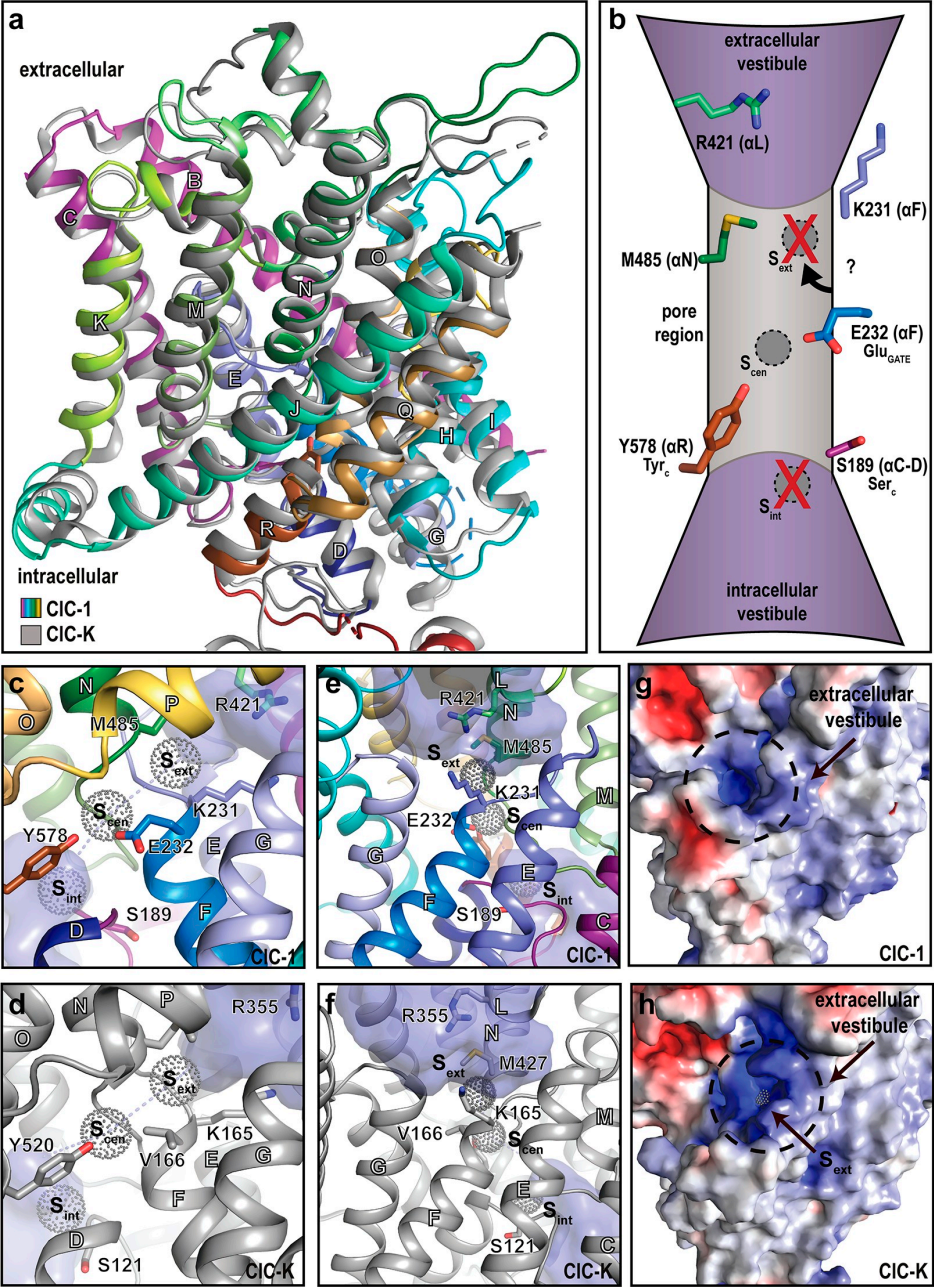
The ion-conducting pathway. Ion transport in CLC proteins depends on extra- and intracellular vestibules and a connecting pore. In CLC transporters, the pore is marked by chloride ion binding sites (s_ext_, s_cen_, s_int_; not directly observed in this work) as well as specific glutamate (Glu_GATE_, or E232; ClC-1 numbering throughout), tyrosine (Tyr_C_, Y578), and serine (Ser_C_, S189) residues. Chloride conductance in voltage-dependent CLC channels such as ClC-1 may involve shuttling (i) to protonated E232-Y578 (s_cen_) from the vestibules - directly (or through a weak s_int_) from the intracellular side and (ii) through K231/R421 to overcome the hydrophobic barrier (including M485) from the extracellular side. (a) Comparison of the transmembrane domains of ClC-1 (colored as in Fig 1D) and ClC-K (gray), respectively. Helices are labelled with white letters throughout. (b) Schematic overview of the chloride permeation pathway with key residues pinpointed. Labels in the parentheses refer to the corresponding helices and the αC–D loop, respectively. (c–f) Side views of the pore region of ClC-1 (panels c and e; colored as in Fig 1D) with equivalent views of ClC-K (panels d and f, shown in gray) [9]. The chloride binding sites are positioned based on the *Escherichia coli* and *Cyanidioschyzon merolae* transporter structures (and are not located in ClC-1 or ClC-K) [8, 14]. The vestibules were calculated using HOLLOW [23] with a probe radius of 1.7 Å and are shown in purple surface. (g–h) Surface electrostatics from the extracellular side of ClC-1 (panel g) and ClC-K (panel h). Red and blue colors represent electronegative and electropositive surfaces, respectively. The chloride binding sites (in CLC transporters) are positioned as in panels c–f. The aperture of the vestibule is narrower in ClC-1 (without visible chloride binding site).

The homodimeric architecture of ClC-1 is reminiscent of that of bovine ClC-K and available structures of CLC proteins from lower organisms (Fig 2A). The monomers consist of membrane-spanning helices and half-helices (αB to αR) with connecting loops (e.g., αB–C, between αB and αC) as well as the CBS domains (Fig 1). Each protomer holds a separate chloride conducting pathway across the membrane, established by a vestibule on either side of the membrane, and an interconnecting narrow and short pore.

In CLC transporters, the Cl^−^ conducting pore (Fig 2B) is marked by distinct Cl^−^ binding sites (denoted s_ext_, s_cen_, and s_int_, respectively, but no Cl^−^ ions are resolved in the current structure), and the constricting Glu232 (of αF, also known as Glu_GATE_; ClC-1 numbering throughout) and Tyr578 (of αR, Tyr_C_) [9]. Furthermore, Ser189 (of αC-D, Ser_C_) is located in the vicinity of the pore (Fig 2B, 2C and 2E and S5A Fig). In ClC-1, voltage-dependent gating is established by Glu_GATE_, which is perhaps being displaced by competing Cl^−^ ions and/or protonation. In contrast, in voltage-independent ClC-K channels, Glu_GATE_ is replaced by a valine, and, indeed, substitutions of Glu_GATE_ with uncharged residues render ClC-1 similarly voltage independent [24]. Unfortunately, the Glu_GATE_ side chain is not visible in our cryo-EM density maps (S4 Fig), but carboxylate groups of interacting acidic residues are known to be frequently undetectable using cryo-EM due to radiation damage. A similar orientation of the side chain as observed in ClC-K would be in agreement with Cl^−^ passage through a maintained s_cen_, as a concomitant adaptation of αR significantly shifts the position of Tyr_C_ and thus maintains the Glu_GATE_-Tyr_C_ distance (Fig 2B–2F and S5G Fig). However, we cannot exclude that the side-chain of Glu_GATE_ is buried deep into the hydrophobic pocket established by Phe279, Phe288, and Phe484 (S5H Fig).

The pore aperture of the extracellular vestibule is constricted by a hydrophobic barrier with Met485 (Met427 in ClC-K), but in contrast to ClC-K, the gate opening is also controlled by Lys231 (of αE–F) and Arg421 (of αL) (Fig 2B–2F) that may orchestrate Cl^−^ permeation to or from the extracellular environment [25–27]. This difference can be attributed to αE–F, with its Glu_GATE_ and Lys231 adopting a more CLC-transporter–like configuration because this loop is considerably shorter than in ClC-K, alongside a side-chain reorientation of Arg421 (Fig 2C–2F). We also observe a structural adjustment on the intracellular side of the pore, with αC–D being displaced as compared to the corresponding loop in ClC-K. This rearrangement opens the vestibule even deeper toward Glu_GATE_ (Fig 2C–2F), providing intracellular access beyond the s_int_ site present in CLC antiporters and suggesting that no tight Cl^−^ binding occurs on the intracellular side, in agreement with electrophysiological data [28]. The wider intracellular vestibule of the CLC channels, as compared to the transporters, has been proposed to allow for the higher Cl^−^ conductance in channels, lowering the kinetic barrier between s_cen_ and the cytosol [9]. We note that the vestibule width of ClC-1 is similar to that of ClC-K at Ser_C_, with the side chain of this residue being positioned away from the Cl^−^ permeation pathway in both channels, establishing the Ser_C_ location as another of the distinguishing features between CLC channels and transporters.

It remains obscure whether the channel has been captured in the open configuration, a priori induced by the experimental conditions (0 mV, 100 mM Cl^−^). Molecular dynamics simulations of the ClC-1 structure suggest that Cl^−^ from the intracellular side spontaneously interacts with Glu_GATE_ upon protonation of its side chain but that free energy is required to complete the passage across the membrane (S6 Fig). We anticipate that Glu_GATE_ and the Lys231–Arg421 constricting interactions attenuate chloride flux, in agreement with the smaller conductance of ClC-1 versus ClC-K [2, 3], and we cannot exclude that Cl^−^ shuttling occurs directly between protonated Glu_GATE_ and Lys231 across the Met485 barrier (Glu_GATE_ overlays s_ext_ in some CLC transporters [8, 14, 29]); chloride interaction with the latter may be unfavorable, however.

**Fig 3:**
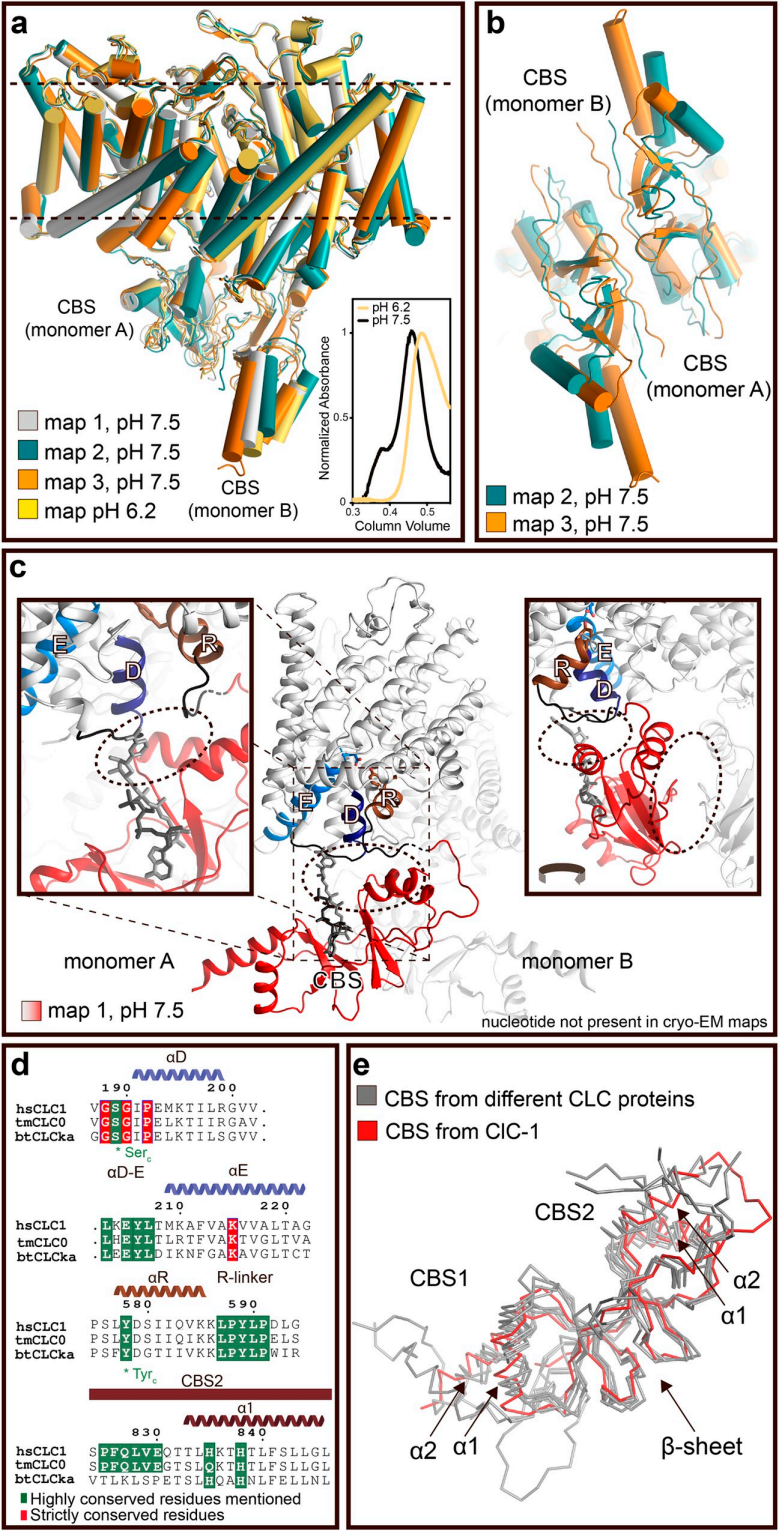
Slow gating of ClC-1 is regulated by pH and nucleotide binding through the CBS domains. (a, b) CBS domain flexibility observed at pH 7.5 but not pH 6.2 in different cryo-EM maps. The most different ones represent Map 2 and Map 3, which were calculated from the pH 7.5 data set (panel b represents a close view; see also S7 and S8 Figs). The inset of panel a represents size-exclusion chromatography profiles of ClC-1 at pH 7.5 and 6.2. The protein peak at pH 6.2 is shifted toward a higher retention volume indicating a more compact ClC-1 (see also S9 Fig). (c) Arrangement of important structural elements in the ClC-1 structure, including αD, αF, αR, and αD−E loop as well as the linker after αR, α1, and α2 in the CBS2 domain. The colors are as in Fig 1D. The ATP molecule (black) is positioned based on the location in ClC-5 (pdb-id 2J9L) [13], and the NAD (gray) placed by exploiting the same base moiety as for ATP. Note that no nucleotide is visible in our structural data, and therefore the observed structural shifts may relate to the lower pH only (the nucleotide is placed based on structures of isolated CBS domains; see panel e). Dotted areas represent putative sites for communication between CBS domains of different monomers and with the transmembrane domain. Details of the interaction network between the CBS domains and the transmembrane domain remain elusive, due to the intermediate resolution of the maps. Helices are labelled with white letters throughout. (d) Reduced sequence alignment of selected putative communication regions between the CBS and transmembrane domains (see S10 Fig for complete alignment). (e) Maintained overall fold of experimentally structurally determined CBS domains of different CLC members, including ClC-K (pdb-id 5TQQ) [9], CLC-0 (pdb-id 2D4Z) [30], CLC-5 (pdb-id 2J9L) [13], and CmClC (pdb-id 3ORG) [14]. ClC-1 is colored in red, the other structures are all in gray. CBS, cystathionine-β-synthase; cryo-EM, cryo-electron microscopy; NAD, nicotinamide adenine dinucleotide; pdb-id, Protein Data Bank ID.

The molecular mechanisms that govern slow gating in CLC proteins remain elusive. It is known that CBS nucleotide binding and low pH inhibit ClC-1 activity by favoring closure of the common gate [19, 29]. Assessment of the 3 major cryo-EM maps obtained in our structural classification (see also S2 Fig and Methods) reveals different arrangements of the CBS domains, suggesting intrinsic domain flexibility at pH 7.5 (Fig 3A and 3B and S7 and S8 Figs). To test this, we determined the structure of ClC-1 also at lower pH (6.2) in the presence of 0.3 mM of the nucleotide nicotinamide adenine dinucleotide (NAD) to unravel the regulation mechanism (S2, S3 and S8 Figs). In these conditions, the CBS domains appear significantly more rigid (in comparison to pH 7.5; Fig 3A and 3B and S7 and S8 Figs). This observation is also supported by ClC-1 size-exclusion chromatography profiles (S9 Fig), with samples at low pH being shifted toward lower molecular weight (more compact). Therefore, the CBS arrangements seem to correlate with slow gating, being rigid at low pH in the presence of nucleotides and more flexible at higher pH in the absence of nucleotides, bringing to mind a mechanism that has been proposed based on electrophysiological data [29]. The complete effects of such putative rearrangements are, however, not demonstrated experimentally by our structures, because they remain closed also at the higher pH (determined from particles in detergent environment).

How then can the Cl^−^ conductance of 2 separate pores be affected by structural shifts of the CBS domains? Examination of the interface between the CBS and the transmembrane domain suggests that CBS2 interacts with αD–E, a loop previously shown to affect slow gating (Fig 3C and 3D) [25, 31]. Nucleotides may also interact directly with the transmembrane domain when bound in the cleft between CBS1 and CBS2 (the latter observed in structures of isolated CBS domains [13]; Fig 3E). It is conceivable that these structural arrangements and the direct physical connection between CBS and αR—all structural elements leading to the Glu_GATE_ constrictions site—allow structural adjustment of the transport pathway and thus chloride conductance regulation (Fig 3C). Such structural effects will be propagated between the monomers via the CBS domains, in agreement with concurrent modulation of the 2 conducting pathways in the dimer [16]. We note that the CBS portions that interact with the transmembrane and the CBS domain of the adjacent monomer are structurally (and at interaction sites also sequencewise; S10 Fig) conserved (Fig 3E), and therefore this may represent a unifying mechanism of slow gating for CLC proteins.

**Fig 4:**
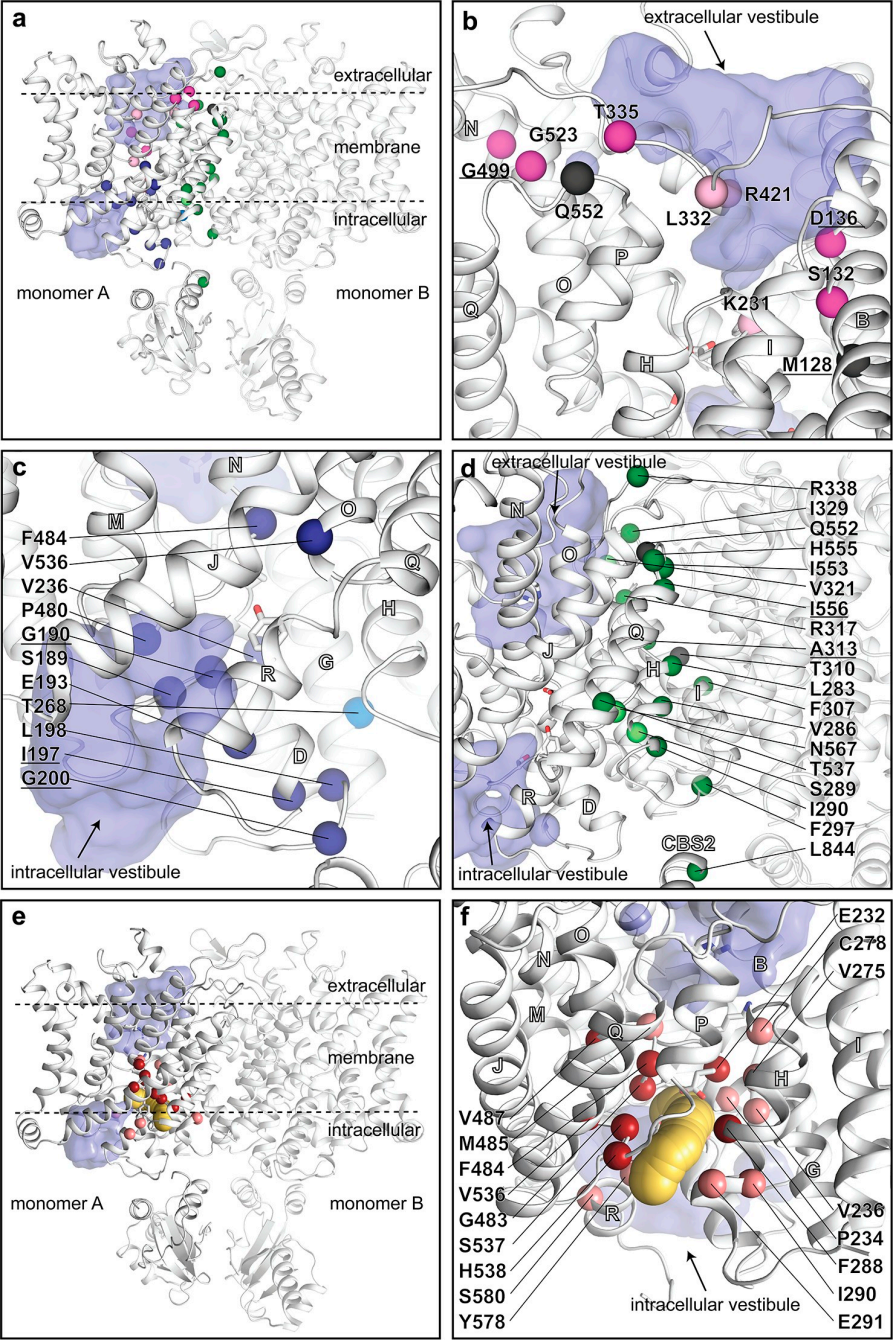
Myotonia-causing mutations and the putative binding pocket of the 9-AC inhibitor. (a–d) Disease-causing and experimental missense mutations in ClC-1. Substitutions that invert (from depolarization to hyperpolarization activated) or shift the voltage dependence are shown in pink (located to the extracellular side) and blue (intracellular vestibule) or green (subunit interface), respectively. Bright colors represent disease-causing (recessive, with a stronger phenotype, but not dominant mutations are underscored), whereas experimental mutations are shown in pale colors. ClC-1 is shown in white and the Cl^−^ vestibules in purple (calculated using HOLLOW as for Fig 2). (a) Overall view with all known disease and selected experimental mutations. We note that mutations of 5 residues that cause recessive myotonia and inward rectification are facing the extracellular vestibule; 3 located in a row on the same face of on helix B (M128, S132, D136) and 2 being the pore-constricting residues (K231, R421). Therefore, the phenotype may reflect a decreased chloride affinity of an extracellularly accessible site. (b) Close view of mutations that invert voltage dependence. Helices are labelled with white letters throughout. (c) Close view of mutations that shift voltage dependence (located at the intracellular vestibule). (d) Close view of mutations that shift voltage dependence (located at the monomer:monomer interface). (e−f) A putative binding pocket of 9-AC. Residues known to affect binding of 9-AC are highlighted as spheres (red for strong effect and pink for minor) and overlay a CAVER calculated pathway (shown in yellow) that stretches from the intracellular membrane interface to Glu_GATE_. The clustering hints at a suitable target point for future rational drug-design efforts (see also alternative view in S11 Fig). CBS, cystathionine-β-synthase; 9-AC, 9-anthracene-carboxylic acid.

ClC-1 defects cause recessive (Becker type) or dominant (Thomsen type) myotonia congenita, typically associated with complete disruption of channel function or with a dominant negative effect in heterodimeric wild-type (WT)-mutant complexes [7], respectively. Our structure now allows mapping of such (or other experimental) ClC-1 substitutions for evaluation of structure–function–disease and -phenotype relationships (Fig 4). Several dominant and recessive mutations induce an alteration of the overall gating from depolarization to hyperpolarization activated, yielding a similar intracellular Cl^−^-sensitive gating as described for ClC-2 [32]. Therefore, the different gating profiles of ClC-1 and ClC-2 likely do not necessitate major structural differences. These residues are generally surface exposed and localized to the extracellular half, including the vestibule and the pore-constricting residues Lys231 and Arg421 (Fig 4B) [26, 27, 32–35]. In contrast, many dominant mutations exert a “shift” of the common gate to open probability to positive voltages, leading to significant reduction of g_Cl_ at the physiological membrane potential [36]. Such mutations cluster primarily at the dimer interface and in the intracellular vestibule and pore region (Fig 4C, and 4D and S5D Fig). One is located in CBS2, close to the membrane domain, in agreement with the above-mentioned mechanism of slow-gating regulation exerted via CBS2. Residues that affect binding of one of the most commonly used ClC-1 inhibitors, the lipophilic 9-anthracene-carboxylic acid (9-AC), are all buried into a CAVER [37]-computed membrane-embedded cavity on the intracellular side that stretches to Glu_GATE_, in agreement with the intracellular mechanism of action proposed for this compound (Fig 4E and 4F and S11 Fig) [24]. Because this pocket is lined by multiple hydrophobic and a few negatively charged residues, it is unlikely to allow chloride conductance (proton access is possible) but rather 9-AC–induced interference of flux across Glu_GATE_ and may thus represent a suitable site for future drug-discovery efforts.

## Discussion

In summary, we report the molecular structure of Cl^−^-conducting human ClC-1, sharing an overall fold similar to other CLC proteins, with a narrow connecting pore and positively charged vestibules attracting Cl- ions similar to CFTR [38]. The structure exhibits several unique features, including shifts in the central Glu_GATE_-Tyr_C_ pair, a more closed extracellular vestibule, and a wider penetration profile from the intracellular side, the latter representing a distinct feature of CLC channels separating them from transporters. We propose a model for adenine nucleotide and pH regulation of the common gate via CBS2 and the intracellular loops congruent with previous functional data. Overall, these findings significantly increase our understanding of Cl^−^ conductance in physiology and open new opportunities for biomedicine. For example, the positively charged constriction of the extracellular vestibule and the putative 9-AC pocket may serve as favorable target sites for stimulators or inhibitors from outside or inside the cell, respectively.

During the course of the preparation of this manuscript, the structure of human ClC-1 was reported by another group [39]. The ClC-1 structures display only limited differences despite that different overproduction hosts were exploited. The authors detected a similar putative 9-AC binding pocket (the alternative pathway) and conformational flexibility in the CBS region (determined at pH 7.4), in agreement with our findings. We anticipate that the pH-dependent conformational changes reported here—in conjunction with mutational efforts using, e.g., single-channel recordings, as for the first time demonstrated in this work, will allow for more refined studies to further resolve the mechanism of slow-gating in CLC proteins.

## Methods

### Recombinant expression construct

Yeast codon-optimized cDNA encoding human ClC-1 (UniProt accession P35523) was purchased from Genscript (Genscript, USA). cDNA was inserted into pEMBLyex4 [40] along with yeast-enhanced GFP by homologous recombination to encode ClC-1, followed by a Tobacco Etch Virus (TEV) cleavage site, GFP, and a His_10_ tag. The correct nucleotide sequence of the expression construct was verified by DNA sequencing (Eurofins MWG Operon, Germany).

### ClC-1 expression and purification

Human ClC-1 was produced in the PAP1500 strain [41] grown in computer controlled 15-L bioreactors as previously reported but without addition of any chloride salts (such as NaCl) [42]. Yeast cells were harvested approximately 90 hours after induction of ClC-1 expression.

For crude membrane preparations, approximately 25 g of yeast cells were resuspended in 25 mL lysis buffer (25 mM imidazole [pH 7.5], 1 mM EGTA, 1 mM EDTA, 10% glycerol, 5 mM β-mercaptoethanol) supplemented with protease inhibitors (1 μg/mL leupeptin, pepstatin, and chymostatin, and 1 mM PMFS). Cells were disrupted by addition of glass beads (0.4–0.8 mm) and vortexed in 50-mL Falcon tubes 8 times for 1 minute. The supernatant was collected, and glass beads were washed several times in ice-cold lysis buffer. The cell lysate was centrifuged at 1,000*g* for 10 minutes to remove cell debris. Crude membranes were pelleted from the supernatant by ultracentrifugation at 160,000*g* for 90 minutes; resuspended in a buffer containing 50 mM Tris (pH 7.5), 300 mM NaCl, 10% glycerol, 1 mM PMSF, and EDTA-free protease inhibitors (Sigma); and homogenized in a Potter-Elvehjem homogenizer. Subsequently, membranes were solubilized by adding dodecyl-β-maltoside (DDM) and cholesteryl semi succinate (CHS; from Anatrace) at final concentrations of 1% and 0.33%, respectively, and incubated at 4°C for 3 hours under gentle stirring. Nonsolubilized material was removed by ultracentrifugation at 30,000 rpm for 30 minutes in a Beckman Ti 60 rotor. Ni-beads from 5 mL of slurry (Thermofisher) were incubated with the supernatant for 2 hours under gentle stirring. To prevent unspecific binding, 30 mM imidazole was added. Resin was transferred to a 5-mL Econo column (Bio-Rad) and washed with 10 column volumes of high-salt buffer (50 mM Tris [pH 7.5], 800 mM NaCl, 5% glycerol, 0.4 mg/mL DDM, and 0.04 mg/mL CHS) followed by 10 column volumes of low-salt buffer (50 mM Tris [pH 7.5], 300 mM NaCl, 5% glycerol, 0.4 mg/mL DDM, and 0.04 mg/mL CHS). ClC-1 protein was liberated from the beads by overnight incubating in 10 mL low-salt buffer containing 0.2 mg of TEV protease. Ni-beads were washed twice with 5 mL of low-salt buffer, and all collections were pooled and concentrated to approximately 1 mL using a 100,000 kDa cutoff concentrator device (Sartorius). Amphipol PMAL-C8 (Anatrace) was added to the purified protein at a mass ratio of 1:5 and incubated overnight. To remove DDM, protein was dialyzed overnight against final buffer (20 mM Tris [pH 7.5], 100 mM NaCl, 0.2 mM TCEP) supplemented with 100 mg of SM-2 Bio-Beads (Bio-Rad). The protein-amphipol complex was applied to a Superdex-200 column equilibrated with final buffer. Peak fractions were collected and concentrated to approximately 0.5 mg/mL. For the low pH samples, the purification procedure was identical except for using 20 mM BisTris (pH 6.2) (instead of Tris [pH 7.5]) in the final buffer (final protein concentration only reached approximately 0.3 mg/mL due to precipitation).

### Single-channel ion conductance

Single-channel ion current was recorded using 2 separate methods, as follows:

1. **The Nanion Orbit Mini bilayer system.** Lipid bilayers were formed using 10 mM 1.2-diphytanoyl-sn-glycero-3-phosphocoline (DPhPc) and 1 mM cholesterol in n-nonane (Avanti Polar Lipids), and single channels were inserted by addition of purified protein (0.2 μL of 0.59 μg/μl ClC-1 in DDM) to recording solution at the *cis* side of the bilayer (150 μL). Current was recorded at ±150 mV in symmetrical solutions containing 1 M KCl and 10 mM HEPES (pH 6.2 with KOH). Recordings were digitized at 1.25 kHz, low-pass filtered at 160 Hz, and analyzed using Clampfit 10 after 100 Hz digital filtering.
2. **The Nanion Port-a-Patch system.** Giant unilamellar vesicles (GUVs) were made from 5 mM DPhPc and 0.5 mM cholesterol in chloroform by electroformation in 1 M sorbitol using the Vesicle Prep Pro (Nanion Technologies). Purified ClC-1 protein in DDM was mixed with GUVs to a final concentration of approximately 50 ng/mL and incubated overnight at 4°C with SM-2 Bio-Beads (Bio-Rad). Lipid bilayers were formed from the GUVs, and single-channel current was recorded in symmetrical solutions containing 1 M NaCl and 10 mM HEPES (pH 6.2 with NaOH) at ±150 mV for 1 second. Recordings were digitized at 50 kHz, low-pass filtered at 200 Hz, and analyzed using Clampfit 10.7 software (Molecular Devices, San Jose, CA).

### Cryo-EM sample preparation and data collection

Cryo-EM grids were prepared with the Vitrobot Mark IV (FEI) operated at 100% humidity at 4°C. Immediately prior to sample vitrification, Quantifoil 1.2/1.3-μm holy carbon grids were glow-discharged with Easyglow (TedPella), and fluorinated fos-choline-8 (Anatrace) was added to the protein sample to a final concentration of 3 mM, which was an essential step for producing good quality thin ice. For each grid, an aliquot of 3.5 μL was applied and incubated for 20 seconds inside the Vitrobot. Blotting time was set to 2.5 seconds with 2 seconds of drain time. The low pH sample was treated identically, except for incubation with 0.3 mM NAD before freezing (and that no fluorinated fos-choline-8 was added to obtain one of the pH 6.2 data sets). Cryo-EM data sets were collected on a Titan Krios electron microscope (FEI) operating at 300 keV with a Gatan K2 Summit direct electron detector attached to a Gatan imaging filter (GIF). Movies were recorded under super-resolution counting mode at a pixel size of 0.535 Å and a dose rate of 0.876 e/pixel/frame for a total of 60 frames. The total electron dose was 45 electrons per Å^2^ per movie for 9 seconds.

### Image processing and 3D reconstruction

Cryo-EM movies were first gain-corrected and 2× binned to a final pixel size of 1.07 Å. Dose-weighted and nondose-weighted summed micrographs were generated with MotionCorr2 [43] using all frames except the first one. Defocus values were calculated with the nondose-weighted micrographs using Gctf [44]. Next, image processing was conducted using dose-weighted micrographs with the predetermined defocus. Template-free particle picking was done using Kai Zhang’s Gautomatch software (https://www.mrc-lmb.cam.ac.uk/kzhang/Gautomatch). All following processing steps were done in Relion 2.0 [45] using a box size of 288 pixels.

For the pH 7.5 data set, a total of 594,609 auto-picked particles from 4,475 micrographs with a defocus range of −1.0 to −3.0 μm were subjected to several rounds of reference-free 2D classification to remove defective particles. The selected 477,729 particles were sorted using 3D classification. Selected classes were refined using masks, either with the complete protein excluding the amphipol belt or with the membrane domain only. Multiple cryo-EM density maps were calculated demonstrating structural heterogeneity of the protein.

3D classification of particles into 5 classes provided the best class consisting of 176,871 particles (representing more than 37% of all particles). A soft mask covering the entire protein without amphipol belt yielded a map with an overall resolution of 4.00 Å, and a tighter mask only containing the membrane domain resulted in map with resolution of 3.63 Å. To further investigate the structure heterogeneity in the cytoplasmic domain, the 2D selected particles were first refined, and then the refined per-particle parameters were applied for 3D classification, only performing local angular searches within ±10 degrees. This local 3D classification resulted in 9 classes, and the 2 major classes differed primarily in the cytoplasmic domain. Refinement of these 2 classes, each representing approximately 15% of all selected particles, yielded overall map resolutions of 4.34 Å and 4.28 Å, respectively.

For the pH 6.2 data set collected with fluorinated fos-choline-8, 552,914 particles were autoselected from 4,119 motion-corrected micrographs, and 300,572 particles were selected after 2D classification for further processing; 3D classification into 5 classes generated the best class, which eventually was refined to a final resolution of 4.47 Å. Combination of the data collected at pH 6.2 with and without fluorinated fos-choline-8, and a similar local angular search strategy as for the pH 7.5 data set, generated a final map of 4.2 Å of the best class (based on approximately 30% of the total particles).

C2 symmetry was applied for all classification procedures, and all maps were sharpened with a B-factor of −100 Å^2^. Local resolution was calculated using the postprocessed map, and the map was filtered according to the local resolution and used for model building.

### Model building and refinement

The initial model was generated using the SWISS-MODEL online server and the ClC-K structure [9] (PDB-ID 5TQQ) as a template. The model was first fitted into the cryo-EM density map and later manually built in COOT [46]. The 3.6 Å membrane domain density map was sufficient for building the entire membrane domain (residues 115 to 589) with only 1 loop missing (residues 254–261). The built model was refined using phenix.real_space_refine of the Phenix software package [47]. C2 symmetry was imposed during the refinement by using strong non-crystallographic symmetry (NCS) restraints. Secondary structure restraints and Ramachandran restraints were also imposed during refinement.

The resolution and connectivity of the cytoplasmic domain was insufficient for de novo model building. Instead, a homology model based on the available structure of the CBS domains of ClC-0 (PDB-ID 2D4Z [30]) was generated and docked into different maps. The refinement of the cytoplasmic domain was conducted by local grid minimization, model morphing, and simulated annealing implemented in the phenix.real_space_refine software [47]. To prevent overfitting, the map resolution was restricted to 5 Å, the local resolution of the cytoplasmic domains as determined by Relion postprocessing. After model building, the models were trimmed to only include the minimal CBS architecture, consisting of 2 helices and a β-sheet. The quality of the models were validated assessed using Molprobity [48] (see S1 Table for statistics). All figures except for Fig 3A and 3B were generated using the model based on the 4.0 Å (Map 1).

### MD simulations

The ClC-1 dimer with Glu232 either protonated or deprotonated was inserted into a palmitoyloleoylphosphocholine (POPC) membrane, and CHARMM36 force field parameters [49, 50] were generated using CHARMM-GUI [51]. The simulations were performed using the GROMACS 2016.4 simulation software [52]. Each system was energy minimized and equilibrated in a stepwise manner using 25-ps NVT simulations with decreasing restraints on the protein and lipid heavy atoms. In these simulations, a 1-fs time step was used and the temperature was maintained at 310 K with a Berendsen temperature-coupling scheme [53]. The following set of NPT simulations further released heavy-atom restraints for 0.1 ns, 10 ns, and 10 ns, respectively. Here, a 2-fs time step was used and the pressure was kept constant at 1 bar using a Berendsen pressure barostat [53]. In a 100 ns production simulation, all atoms were unrestrained, and the temperature and pressure coupling schemes were Nose-Hoover [54, 55] and Parrinello-Rahman [56, 57], respectively. The GROMACS pull code with a force constant of 1,000 kJ mol^−1^ nm^−2^ was applied for 300 ps to the Cl^−^ ion in closest vicinity of Glu232 in 1 monomer. The pull rate was 0.1 Å per ps, and the pull force was directed along the vertical axis of the membrane. The potential of mean force (PMF) was calculated using umbrella sampling from 1 Å windows along the ion path. The figures were generated using VMD software[58].

## Supporting information

S1 Fig Single-channel recordings of purified ClC-1 channels. (a) DDM-solubilized protein was incorporated into planar lipid bilayers consisting of 10 mM DPhPc and 1 mM cholesterol dissolved in n-nonane. Single-channel activity was measured using symmetrical solutions containing 1 M KCl and 10 mM HEPES (pH 6.2) at holding potentials of ±150 mV in the Orbit Mini system (Nanion Technologies). Openings and closings of the incorporated channels are marked, and zero current is indicated by blue lines. (b) DDM-solubilized protein was incorporated into GUVs consisting of 10 mM DPhPc and 1 mM cholesterol, and planar lipid bilayers were formed on an NPC-1 chip using symmetrical solution containing 1 M NaCl and 10 mM HEPES (pH 6.2). Single channel currents were recorded at ±150 mV using Port-a-Patch system (Nanion Technologies). Openings and closings of the incorporated channels are marked, and zero current is indicated by blue lines. (c) Amplitude histogram of single channel recordings obtained at −150 mV under same conditions as in panel b. The distribution of amplitudes was fitted with the sum of 3 Gaussian distributions. Single-channel conductance was calculated to 4.0 ± 0.2 pS (*n* = 21) for recordings obtained in 1 M NaCl and to 3.5 ± 0.1 pS (*n* = 90) for recordings obtained in 1 M KCl. The calculations were based on >3 independent experiments. (d) Single-channel recordings obtained at +200 mV using similar experimental conditions as in panel b, but in the absence and presence of 100 μM the chloride channel inhibitor 9-AC. The channel activity could be recovered after washout of 9-AC. The shown traces are representative of 3 independent experiments. It should be noted that reconstituted ion channels may incorporate with random orientation into the membrane. Therefore, the applied voltage is not necessarily reflecting the direction of the physiological membrane potential, and single-channel rectification properties of ClC-1 may not be correctly reproduced. The large chloride concentration employed (1 M) likely leads to complete opening of the fast gate, explaining why the double-barrelled appearance of ClC-1 is not apparent. Taken together, the measurements in the presence of NaCl or KCl suggest a Cl^−^-dependent single-channel activity resulting from ClC-1. This is further supported by the fact that the current could be totally inhibited by 9-AC. We also note that the ClC-1 overproducing yeast cells were unable to thrive in standard media containing 1.7 mM NaCl and that minimal media without chloride was required for yeast growth and protein production. The underlying data for S1C can be found in S1 Data. CLC, chloride channelGUV, giant unilamellar vesicle; 9-AC, 9-anthracene-carboxylic acid.
(TIF)

S2 Fig Cryo-EM image processing for the pH 7.5 and 6.2 data sets. (a) The 4 maps (Maps 0–3, respectively) generated using the pH 7.5. Maps 1–3 represent the overall structure refined by applying a mask that only covers the protein part without the amphipol belt but with differences in the cytoplasmic CBS domains. Map 0 represents the membrane domain map derived from focused refinement covering the membrane domain only. Map 1 with overall resolution 4.0 Å was generated by applying a mask covering the entire protein excluding the amphipol belt. After 3D refinement with a membrane domain mask, Map 0 with a resolution of 3.63 Å was obtained. Maps 2 and 3 were produced by 3D refinement of 2 major classes obtained from 3D classification using a local angular search strategy based on the model generated from 477,729 particles by 3D refinement directly (see Methods for further details). (b) pH 6.2 is suboptimal for ClC-1, leading to partial aggregation during purification and freezing. Hence, the collected data set at pH 6.2 is of less quality than that collected at pH 7.5. To obtain the pH 6.2 structure, we combined 2 data sets: (i) a data set collected with fluorinated fos-choline-8 (as the pH 7.5 data set) processed to an overall resolution of 4.47 Å (derived from 34.2% of the particles following 3D classification into 5 classes; we did not identify secondary structure features for the remaining 4 classes, suggesting that there is a large fraction of low-quality particles in the data), and (ii) a second data set without fluorinated fos-choline-8. The second pH 6.2 dataset yielded nonoptimal ice thickness but provided views that were not observed in the first one. The final map derived from combination of these two data sets, following 3D classification with a local angular search strategy as for the pH 7.5 data set, produced 5 classes, of which the best was refined to an overall resolution of 4.2 Å. This class represents 30.6% of the particles, of which 76,881 particles were from the first data set and 49,221 particles were from the second data set. New orientation that was not observed in the first data set is highlighted with a red square in the lowest image. The density of the cytoplasmic CBS domains are better resolved at pH 6.2 compared to the pH 7.5 data set (see also S7 Fig). The maps are all contoured at level σ = 0.013 in Chimera. CBS, cystathionine-β-synthase; cryo-EM, cryo-electron microscopy.
(TIF)

S3 Fig Evaluation of the local resolution of the cryo-EM maps. From top to bottom: (a) Map 0, (b) Map 1, (c) Map 2, (d) Map 3 (all of the pH 7.5 data set), and (e) the final map from the pH 6.2 data sets. See S2 Fig for further information regarding the generated maps. From left to right, Euler angle distribution, FSC, masks exploited for the refinement evaluations, and color-coded local resolution distribution calculated by Relion in two different views (the maps are contoured at level σ = 0.03 in Chimera, except for Map 0, which is at level σ = 0.044). The angular distribution plots suggest a high degree of anisotropy. Note that more density features and better connectivity are observed for the CBS domains in the low pH structure. CBS, cystathionine-β-synthase; cryo-EM, cryo-electron microscopy; FSC, Fourier shell correlation.
(TIF)

S4 Fig Cryo-EM density of selected parts of the ClC-1 membrane region. The helices are colored as in Fig 1D with the maps contoured at level σ = 0.03 in Chimera using Map 0. The modelled Glu_GATE_ (E232) is colored blue and an alternative (but not modeled) orientation is shown in gray. Numbers in parentheses indicate shown residues. cryo-EM, cryo-electron microscopy.
(TIF)

S5 Fig Novel structural features revealed by the ClC-1 structure. Depicted as in Fig 1D and with ClC-K in gray, the new features are highlighted with arrows. (a–f) The αC–D, αE–F, αH–I, αI–J, αL–M, and αN–O loops, respectively. Helices are labeled with white letters throughout. (d) Details of the extracellular αI–J loop, which is targeted by several dominant disease mutations (see also Fig 4). This loop was not observed in the ClC-K and CmClC (PDB-ID 3ORG) [14] structures. Note the short distance between the αI–J loop and R421 of the vestibule, hinting at a role for αI–J in controlling chloride passage. Residue T335 (which was differently placed in a recent homology model [59]) in the αI–J loop is within reach of Q552 in the αO–P loop, possibly providing a communication bridge of extracellular cues to conformational changes at the dimer interface or in the pore region (mutations of both T335 and Q552 cause inward rectification [35]). In ClC-K channels, 2 symmetrically localized inter-subunit regulatory Ca^2+^ binding sites are formed by αI–J loop residues [60, 61]. The corresponding residues in ClC-1 are not oriented in a manner consistent with Ca^2+^ binding. (g, h) Details of E232 (Glu_GATE_) and a possible (not modeled) alternative orientation of its side chain in panel h (see also S4 Fig).PDB-ID, Protein Data Bank ID.
(TIF)

S6 Fig Molecular dynamics simulations suggest that protonated GluGATE (E232) primes ClC-1 with Cl− for ion conductance, but Cl− transfer across GluGATE is nonspontaneous. Simulations were performed in the presence of a POPC membrane and 100 mM NaCl (no gradient). (a–c) Protonated E232 primes ClC-1 with Cl^−^ for conductance without applied force. Observed ion positions (gray spheres) during the course of the simulations with E232 being (a) protonated and (b) deprotonated. Notably, Cl^−^ ions reach R421 from the extracellular side and Glu_GATE_ from the intracellular side. For clarity, only Glu_GATE_, Tyr_C_, and R421 are visualized (as sticks). (c) The number of Cl^−^ ions within 5 Å of Glu_GATE_ in both ClC-1 monomers in simulations with protonated Glu_GATE_ (red) and deprotonated Glu_GATE_ (black). Protonated Glu_GATE_ typically coordinates a single Cl^−^ in the latter half of the simulation. In contrast, Cl^−^ comes into proximity only transiently and is repelled by the negative charge of deprotonated Glu_GATE_. (d–e) Cl^−^ movement across ClC-1 with a protonated E232 from the primed Cl^−^ position at the Glu_GATE_-Tyr_C_ pair (observed in panel a) appears nonspontaneous. (d) Free energy associated with moving (by applying force) Cl^−^ from the primed position to the extracellular side. Positive free energy barriers in the range of 3 kcal/mol indicate that movement along the sampled reaction coordinate is a nonspontaneous process. The distance moved is relative to the Tyr_C_ (Y578) hydroxyl oxygen. (e) The observed (not necessarily native) Cl^−^ (gray spheres) transport pathway exploited for calculating panel d is shown on the overall structure. For clarity, only Glu_GATE_, Tyr_C_, and R421 are pinpointed (sticks). Collectively, these MD simulations suggest that although the determined structure may be closed, Cl^−^ ions may spontaneously penetrate deep into the vestibules from both sides of the membrane. MD, Molecular Dynamics.
(TIF)

S7 Fig Maps of the overall structures and CBS domains, respectively. The maps of the overall structures are shown at level σ = 0.03 in Chimera, whereas those of the CBS domains only are shown at level σ = 0.035. Maps 1–3 of the pH 7.5 data and map of the pH 6.2 data are shown. (a) The overall structures. (b) The CBS domains. CBS, cystathionine-β-synthase.
(TIF)

S8 Fig Details of the CBS shifts. Alternative views of Fig 3 (colored identically). (a) Alternative view of Fig 3B. (b–d) Identical view as in panel a with comparisons of the structures derived from Maps 1–3 (pH 7.5) and the pH 6.2 map (aligned as in Fig 3A), respectively. (e) Identical view as Fig 3C, including the structure determined at pH 6.2. Helices are labeled with white letters throughout. CBS, cystathionine-β-synthase.
(TIF)

S9 Fig Comparison of ClC-1 samples at pH 7.5 and 6.2. (a) Size-exclusion chromatography profiles of ClC-1 at pH 7.5 and 6.2. The protein peak at pH 6.2 is shifted toward a higher retention volume indicating a more compact ClC-1. The peak appearing at 0.7 CV represents the signal from free PMAL-C8 amphipol. (b) Micrographs for the pH 7.5 (left) and 6.2 (middle and right) data sets (with and without F-FC-8, respectively), indicating worse behavior of the pH 6.2 sample. CV, Column volume; F-FC-8, fluorinated fos-choline-8.
(TIF)

S10 Fig Sequence alignment of selected CLC proteins. All human CLC members and structurally determined CLC proteins are displayed. Secondary structure elements are pinpointed, and conserved residues are highlighted in red and green, the latter representing residues relevant for the function of ClC-1 discussed in this work. CLC, chloride channel.
(TIF)

S11 Fig Details of the putative 9-AC pocket. Alternative views of Fig 4E and 4F (colored identically). 9-AC, 9-anthracene-carboxylic acid.
(TIF)

S1 Table Cryo-EM data validation statistics. cryo-EM, cryo-electron micrscopy.
(XLSX)

S1 Data Calculation of single-channel recordings of purified ClC-1. Single-channel conductance was determined based on single-channel current recordings and calculated to 4.0 ± 0.2 pS (*n* = 21) for recordings obtained in 1 M NaCl and to 3.5 ± 0.1 pS (*n* = 90) for recordings obtained in 1 M KCl. Recording solution, command voltage, and current amplitude of the individual recordings are listed.
(XLSX)

## Abbreviations

Arg: arginine
CBS: cystathionine-β-synthase
CFTR: cystic fibrosis transmembrane conductance regulator
CHS: cholesteryl semi succinate;cryo-EM, cryo-electron microscopy
DDM: dodecyl-β-maltoside
GIF: Gatan imaging filter
GUV: giant unilamellar vesicle
Lys: lysine
Met: methionine
NAD: nicotinamide adenine dinucleotide
NCS: non-crystallographic symmetry
Phe: phenylalanine
PMF: potential of mean force
POPC: palmitoyloleoylphosphocholine
TEV: Tobacco Etch Virus
WT: wild type
3D: three-dimensional
9-AC: 9-anthracene-carboxylic acid
