# Supplementary figures and images for: Structure of the human ClC-1 chloride channel
Source: PLoS Biol. 2019 Apr 25;17(4):e3000218. doi: 10.1371/journal.pbio.3000218 (PMC6483157; doi:10.1371/journal.pbio.3000218)

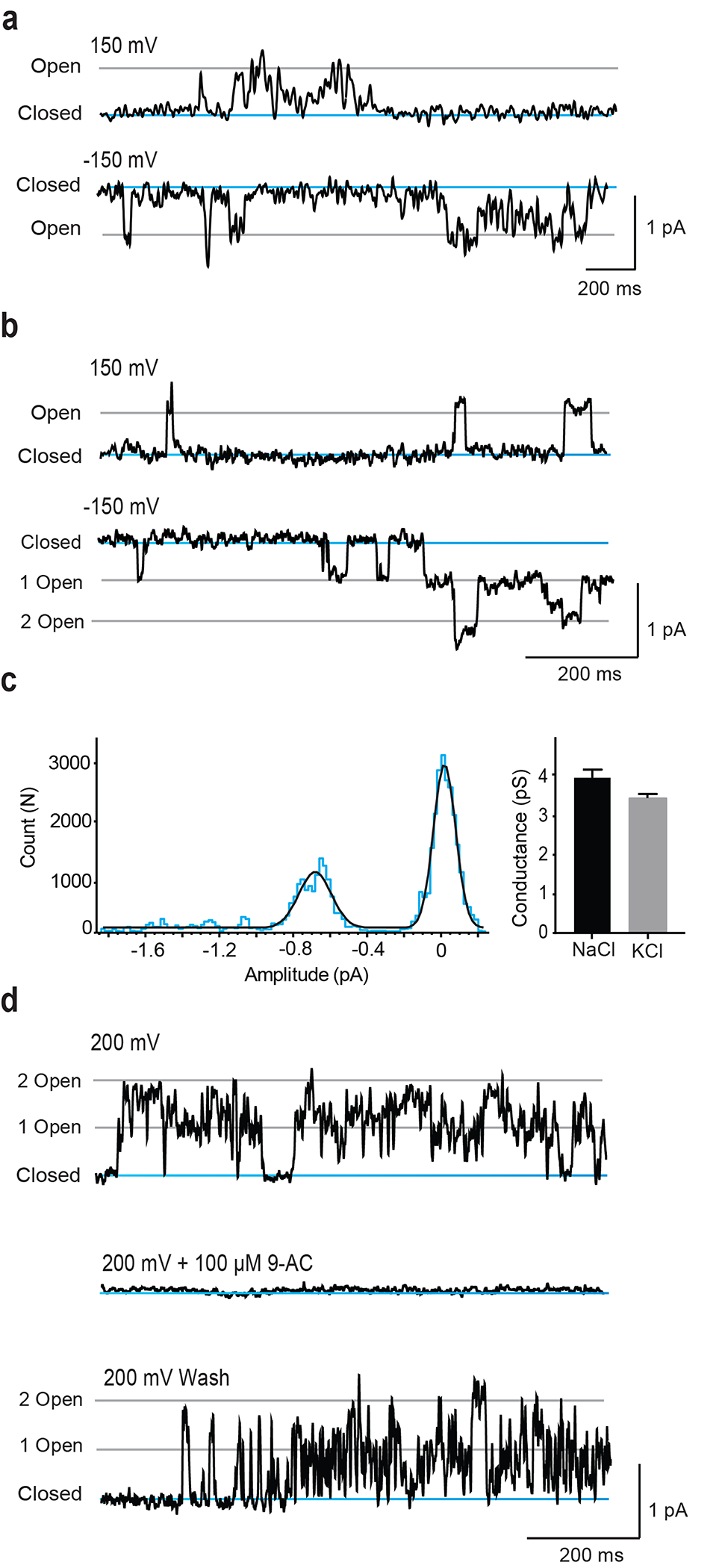

Supplement: S1 Fig — (a) DDM-solubilized protein was incorporated into planar lipid bilayers consisting of 10 mM DPhPc and 1 mM cholesterol dissolved in n-nonane. Single-channel activity was measured using symmetrical solutions containing 1 M KCl and 10 mM HEPES (pH 6.2) at holding potentials of ±150 mV in the Orbit Mini system (Nanion Technologies). Openings and closings of the incorporated channels are marked, and zero current is indicated by blue lines. (b) DDM-solubilized protein was incorporated into GUVs consisting of 10 mM DPhPc and 1 mM cholesterol, and planar lipid bilayers were formed on an NPC-1 chip using symmetrical solution containing 1 M NaCl and 10 mM HEPES (pH 6.2). Single channel currents were recorded at ±150 mV using Port-a-Patch system (Nanion Technologies). Openings and closings of the incorporated channels are marked, and zero current is indicated by blue lines. (c) Amplitude histogram of single channel recordings obtained at −150 mV under same conditions as in panel b. The distribution of amplitudes was fitted with the sum of 3 Gaussian distributions. Single-channel conductance was calculated to 4.0 ± 0.2 pS (n = 21) for recordings obtained in 1 M NaCl and to 3.5 ± 0.1 pS (n = 90) for recordings obtained in 1 M KCl. The calculations were based on >3 independent experiments. (d) Single-channel recordings obtained at +200 mV using similar experimental conditions as in panel b, but in the absence and presence of 100 μM the chloride channel inhibitor 9-AC. The channel activity could be recovered after washout of 9-AC. The shown traces are representative of 3 independent experiments. It should be noted that reconstituted ion channels may incorporate with random orientation into the membrane. Therefore, the applied voltage is not necessarily reflecting the direction of the physiological membrane potential, and single-channel rectification properties of ClC-1 may not be correctly reproduced. The large chloride concentration employed (1 M) likely leads to complete openin [file pbio.3000218.s001.tif]

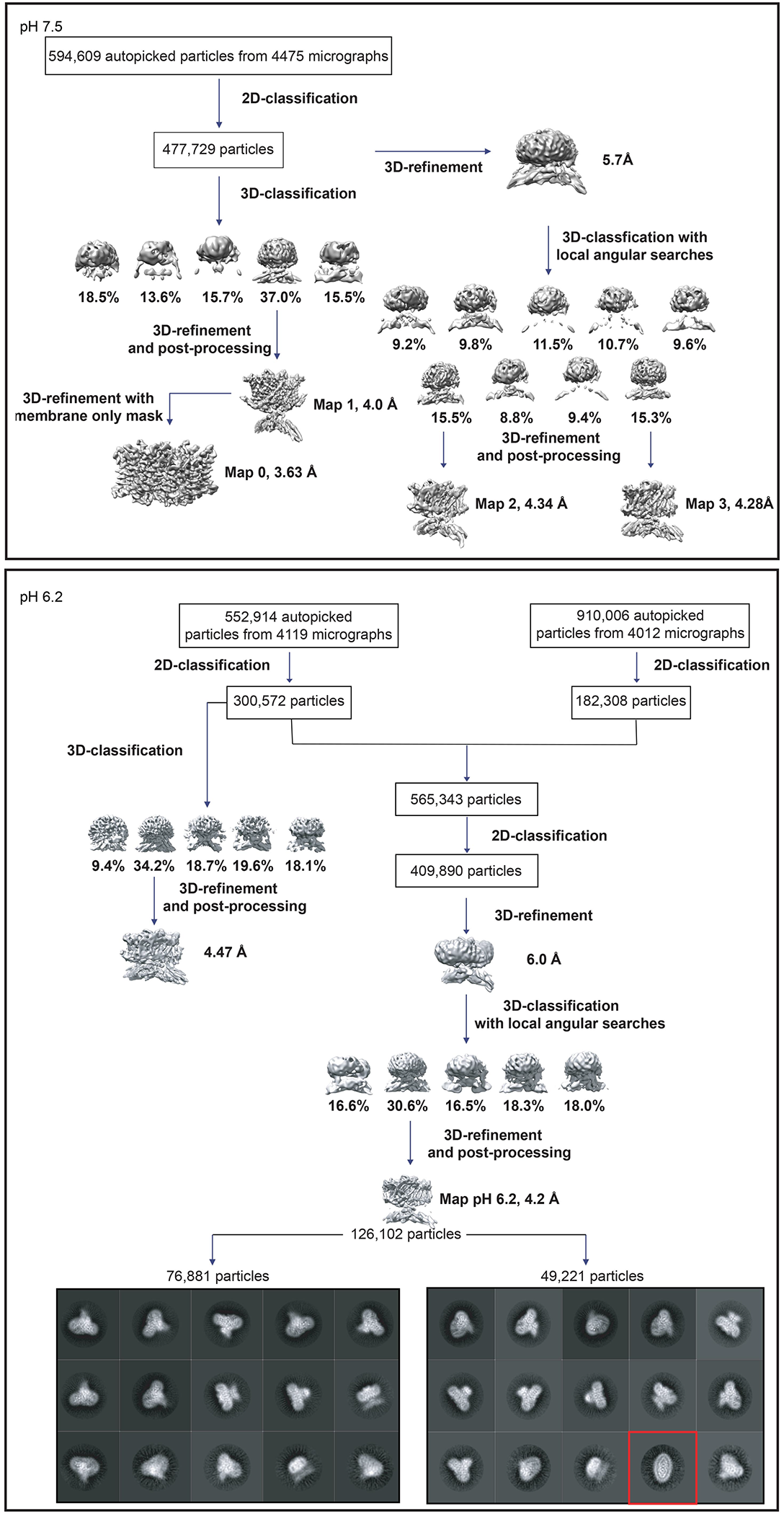

Supplement: S2 Fig — (a) The 4 maps (Maps 0–3, respectively) generated using the pH 7.5. Maps 1–3 represent the overall structure refined by applying a mask that only covers the protein part without the amphipol belt but with differences in the cytoplasmic CBS domains. Map 0 represents the membrane domain map derived from focused refinement covering the membrane domain only. Map 1 with overall resolution 4.0 Å was generated by applying a mask covering the entire protein excluding the amphipol belt. After 3D refinement with a membrane domain mask, Map 0 with a resolution of 3.63 Å was obtained. Maps 2 and 3 were produced by 3D refinement of 2 major classes obtained from 3D classification using a local angular search strategy based on the model generated from 477,729 particles by 3D refinement directly (see Methods for further details). (b) pH 6.2 is suboptimal for ClC-1, leading to partial aggregation during purification and freezing. Hence, the collected data set at pH 6.2 is of less quality than that collected at pH 7.5. To obtain the pH 6.2 structure, we combined 2 data sets: (i) a data set collected with fluorinated fos-choline-8 (as the pH 7.5 data set) processed to an overall resolution of 4.47 Å (derived from 34.2% of the particles following 3D classification into 5 classes; we did not identify secondary structure features for the remaining 4 classes, suggesting that there is a large fraction of low-quality particles in the data), and (ii) a second data set without fluorinated fos-choline-8. The second pH 6.2 dataset yielded nonoptimal ice thickness but provided views that were not observed in the first one. The final map derived from combination of these two data sets, following 3D classification with a local angular search strategy as for the pH 7.5 data set, produced 5 classes, of which the best was refined to an overall resolution of 4.2 Å. This class represents 30.6% of the particles, of which 76,881 particles were from the first data set and 49,221 particles were from the se [file pbio.3000218.s002.tif]

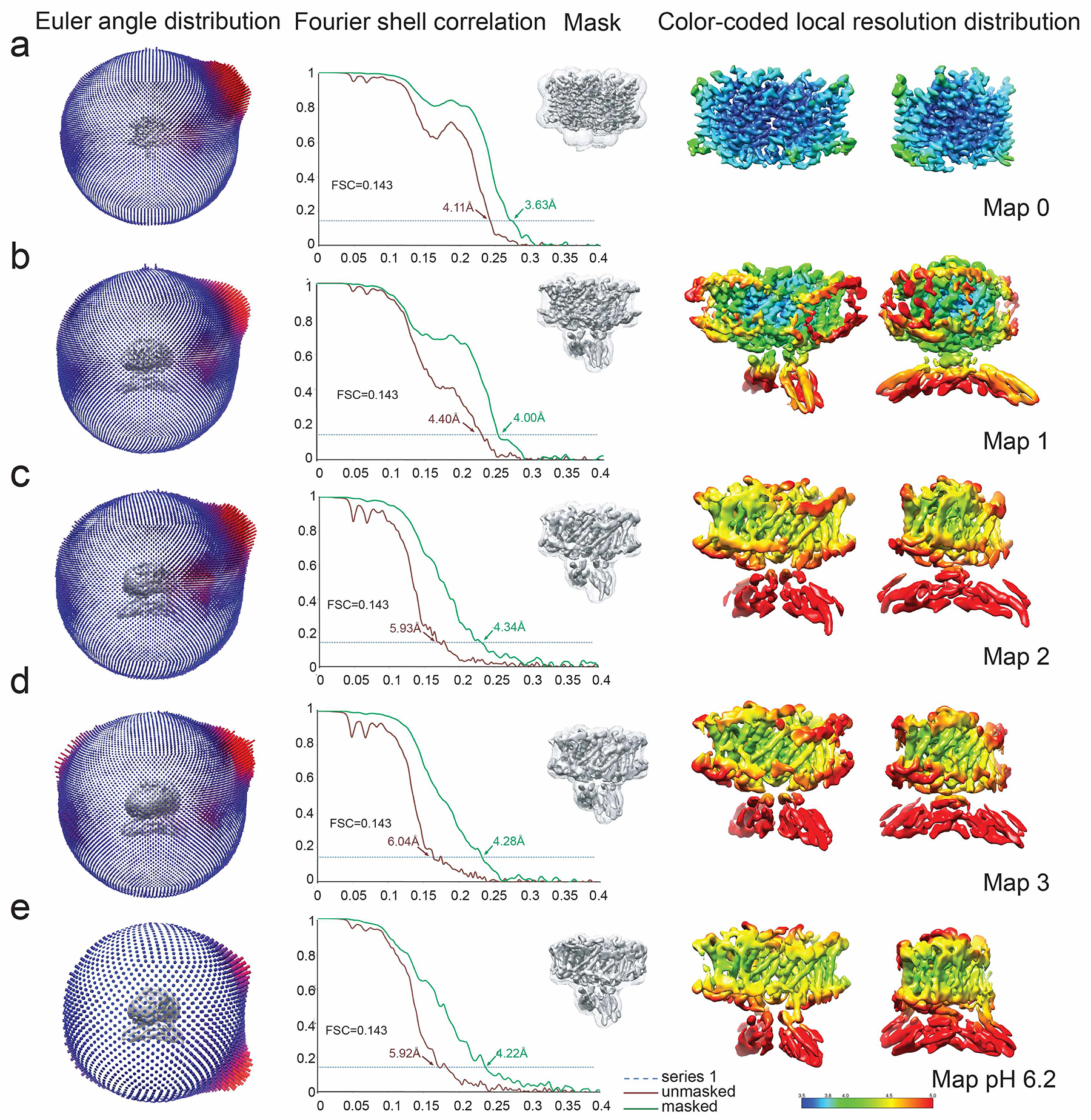

Supplement: S3 Fig — From top to bottom: (a) Map 0, (b) Map 1, (c) Map 2, (d) Map 3 (all of the pH 7.5 data set), and (e) the final map from the pH 6.2 data sets. See S2 Fig for further information regarding the generated maps. From left to right, Euler angle distribution, FSC, masks exploited for the refinement evaluations, and color-coded local resolution distribution calculated by Relion in two different views (the maps are contoured at level σ = 0.03 in Chimera, except for Map 0, which is at level σ = 0.044). The angular distribution plots suggest a high degree of anisotropy. Note that more density features and better connectivity are observed for the CBS domains in the low pH structure. CBS, cystathionine-β-synthase; cryo-EM, cryo-electron microscopy; FSC, Fourier shell correlation. (TIF) [file pbio.3000218.s003.tif]

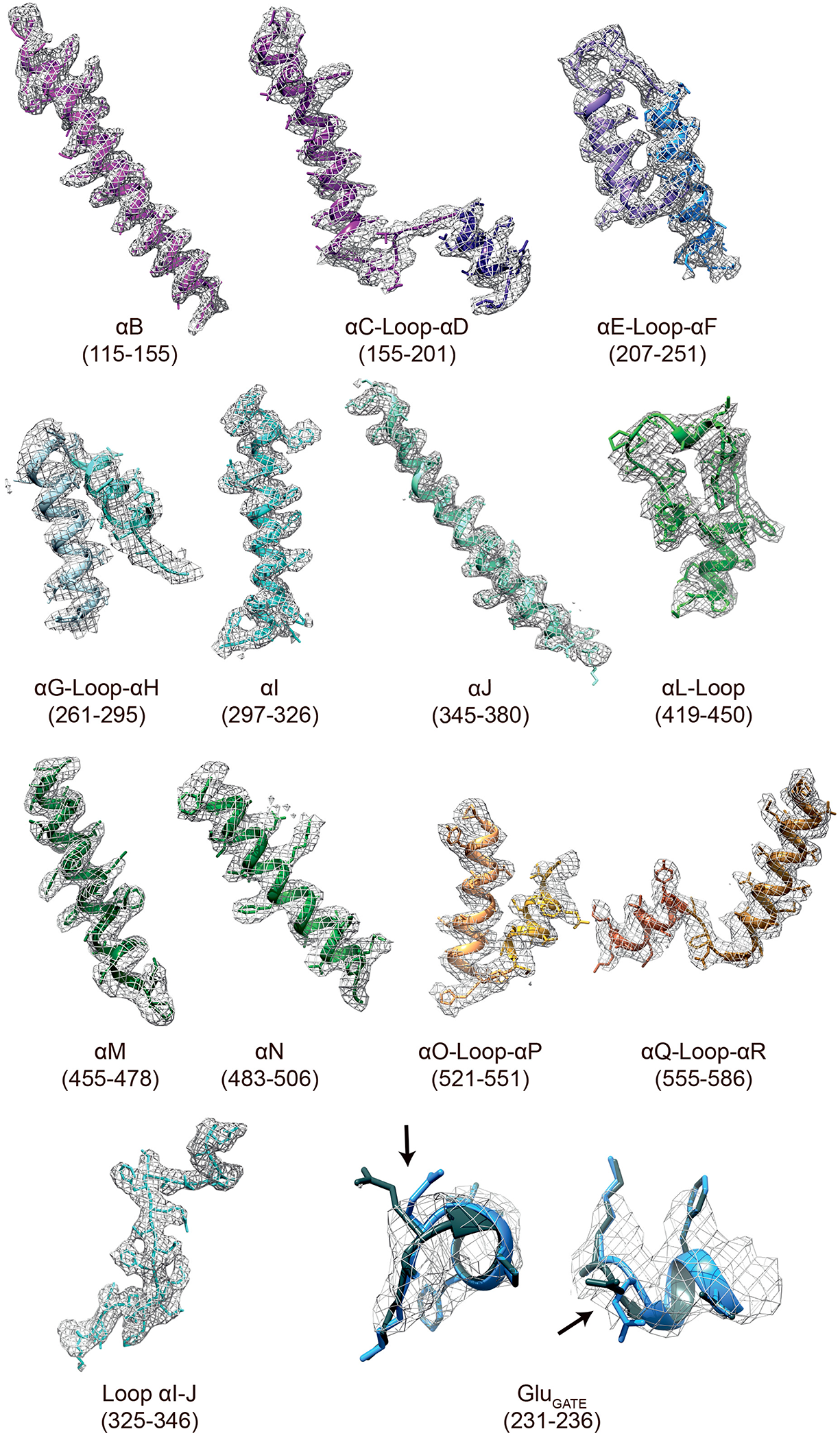

Supplement: S4 Fig — The helices are colored as in Fig 1D with the maps contoured at level σ = 0.03 in Chimera using Map 0. The modelled GluGATE (E232) is colored blue and an alternative (but not modeled) orientation is shown in gray. Numbers in parentheses indicate shown residues. cryo-EM, cryo-electron microscopy. (TIF) [file pbio.3000218.s004.tif]

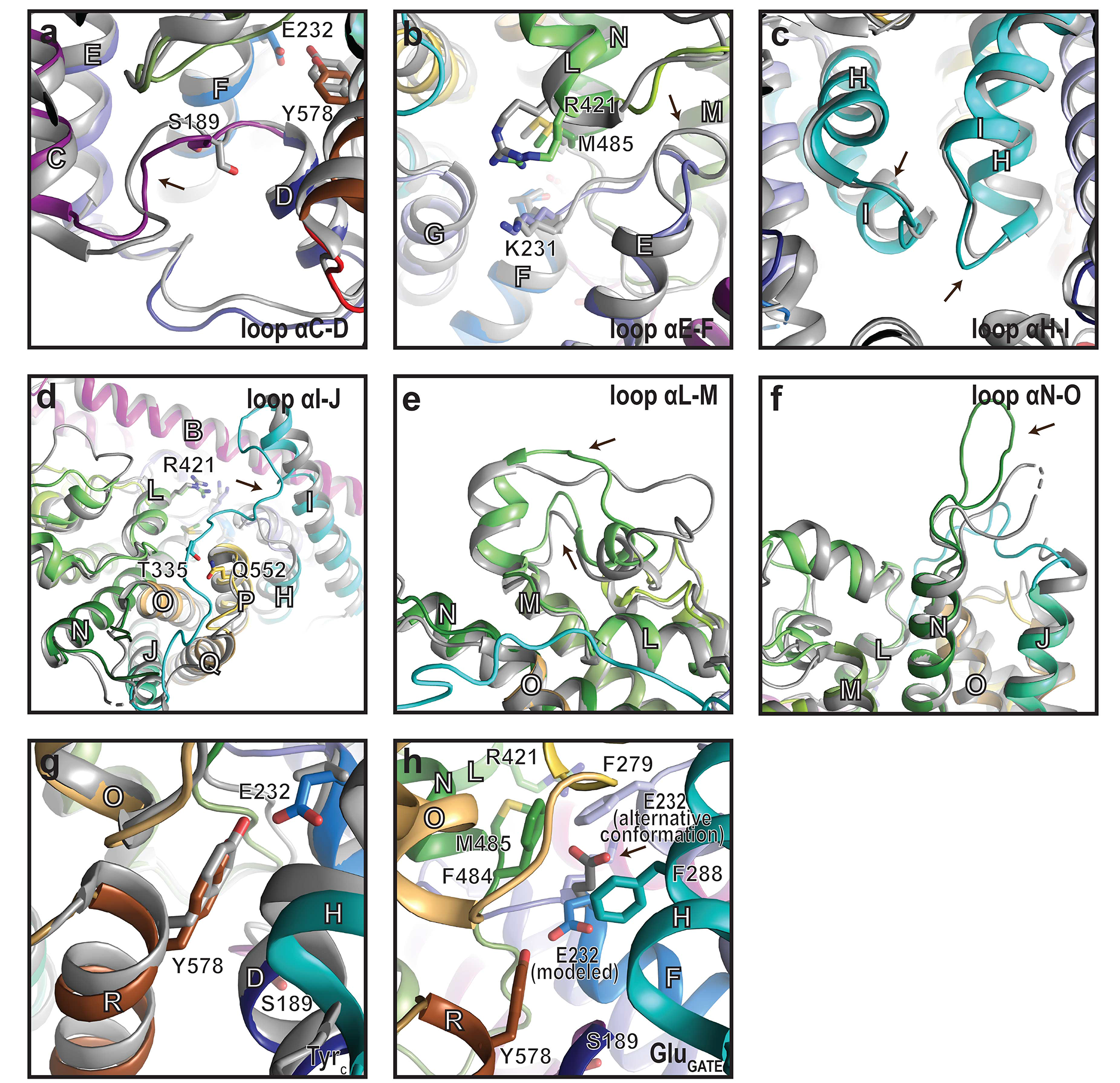

Supplement: S5 Fig — Depicted as in Fig 1D and with ClC-K in gray, the new features are highlighted with arrows. (a–f) The αC–D, αE–F, αH–I, αI–J, αL–M, and αN–O loops, respectively. Helices are labeled with white letters throughout. (d) Details of the extracellular αI–J loop, which is targeted by several dominant disease mutations (see also Fig 4). This loop was not observed in the ClC-K and CmClC (PDB-ID 3ORG) [14] structures. Note the short distance between the αI–J loop and R421 of the vestibule, hinting at a role for αI–J in controlling chloride passage. Residue T335 (which was differently placed in a recent homology model [59]) in the αI–J loop is within reach of Q552 in the αO–P loop, possibly providing a communication bridge of extracellular cues to conformational changes at the dimer interface or in the pore region (mutations of both T335 and Q552 cause inward rectification [35]). In ClC-K channels, 2 symmetrically localized inter-subunit regulatory Ca2+ binding sites are formed by αI–J loop residues [60, 61]. The corresponding residues in ClC-1 are not oriented in a manner consistent with Ca2+ binding. (g, h) Details of E232 (GluGATE) and a possible (not modeled) alternative orientation of its side chain in panel h (see also S4 Fig).PDB-ID, Protein Data Bank ID. (TIF) [file pbio.3000218.s005.tif]

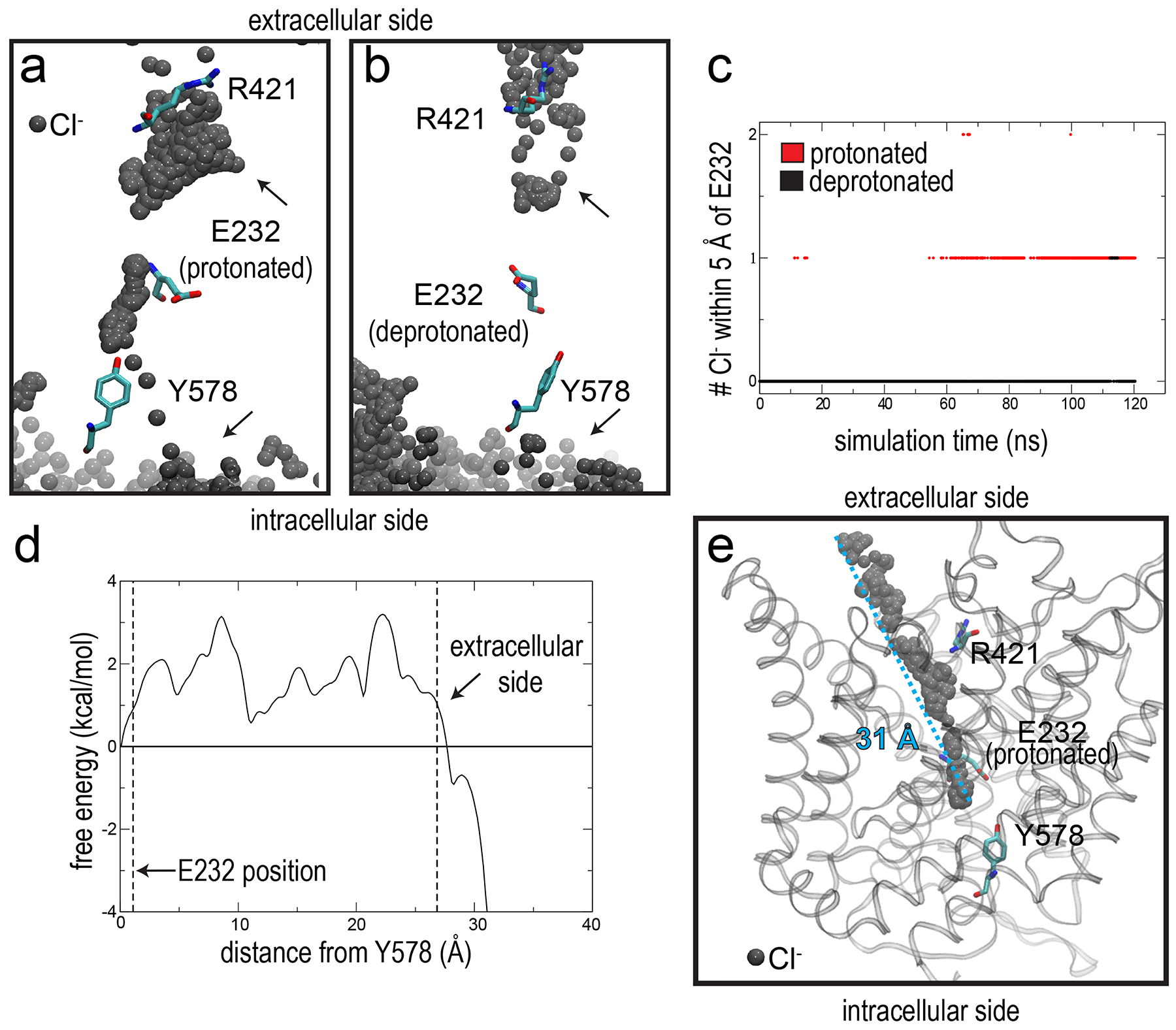

Supplement: S6 Fig — Simulations were performed in the presence of a POPC membrane and 100 mM NaCl (no gradient). (a–c) Protonated E232 primes ClC-1 with Cl− for conductance without applied force. Observed ion positions (gray spheres) during the course of the simulations with E232 being (a) protonated and (b) deprotonated. Notably, Cl− ions reach R421 from the extracellular side and GluGATE from the intracellular side. For clarity, only GluGATE, TyrC, and R421 are visualized (as sticks). (c) The number of Cl− ions within 5 Å of GluGATE in both ClC-1 monomers in simulations with protonated GluGATE (red) and deprotonated GluGATE (black). Protonated GluGATE typically coordinates a single Cl− in the latter half of the simulation. In contrast, Cl− comes into proximity only transiently and is repelled by the negative charge of deprotonated GluGATE. (d–e) Cl− movement across ClC-1 with a protonated E232 from the primed Cl− position at the GluGATE-TyrC pair (observed in panel a) appears nonspontaneous. (d) Free energy associated with moving (by applying force) Cl− from the primed position to the extracellular side. Positive free energy barriers in the range of 3 kcal/mol indicate that movement along the sampled reaction coordinate is a nonspontaneous process. The distance moved is relative to the TyrC (Y578) hydroxyl oxygen. (e) The observed (not necessarily native) Cl− (gray spheres) transport pathway exploited for calculating panel d is shown on the overall structure. For clarity, only GluGATE, TyrC, and R421 are pinpointed (sticks). Collectively, these MD simulations suggest that although the determined structure may be closed, Cl− ions may spontaneously penetrate deep into the vestibules from both sides of the membrane. MD, Molecular Dynamics. (TIF) [file pbio.3000218.s006.tif]

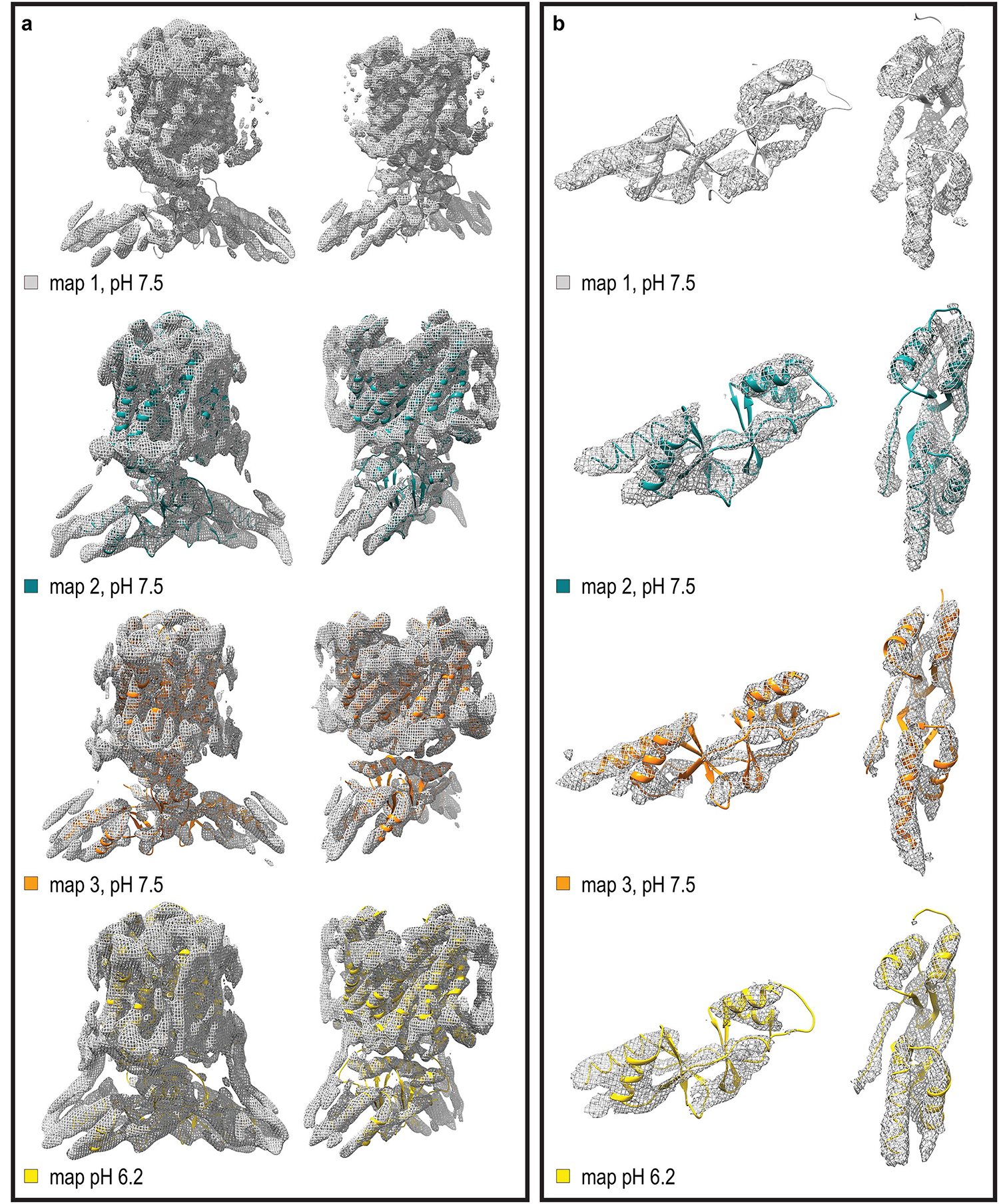

Supplement: S7 Fig — The maps of the overall structures are shown at level σ = 0.03 in Chimera, whereas those of the CBS domains only are shown at level σ = 0.035. Maps 1–3 of the pH 7.5 data and map of the pH 6.2 data are shown. (a) The overall structures. (b) The CBS domains. CBS, cystathionine-β-synthase. (TIF) [file pbio.3000218.s007.tif]

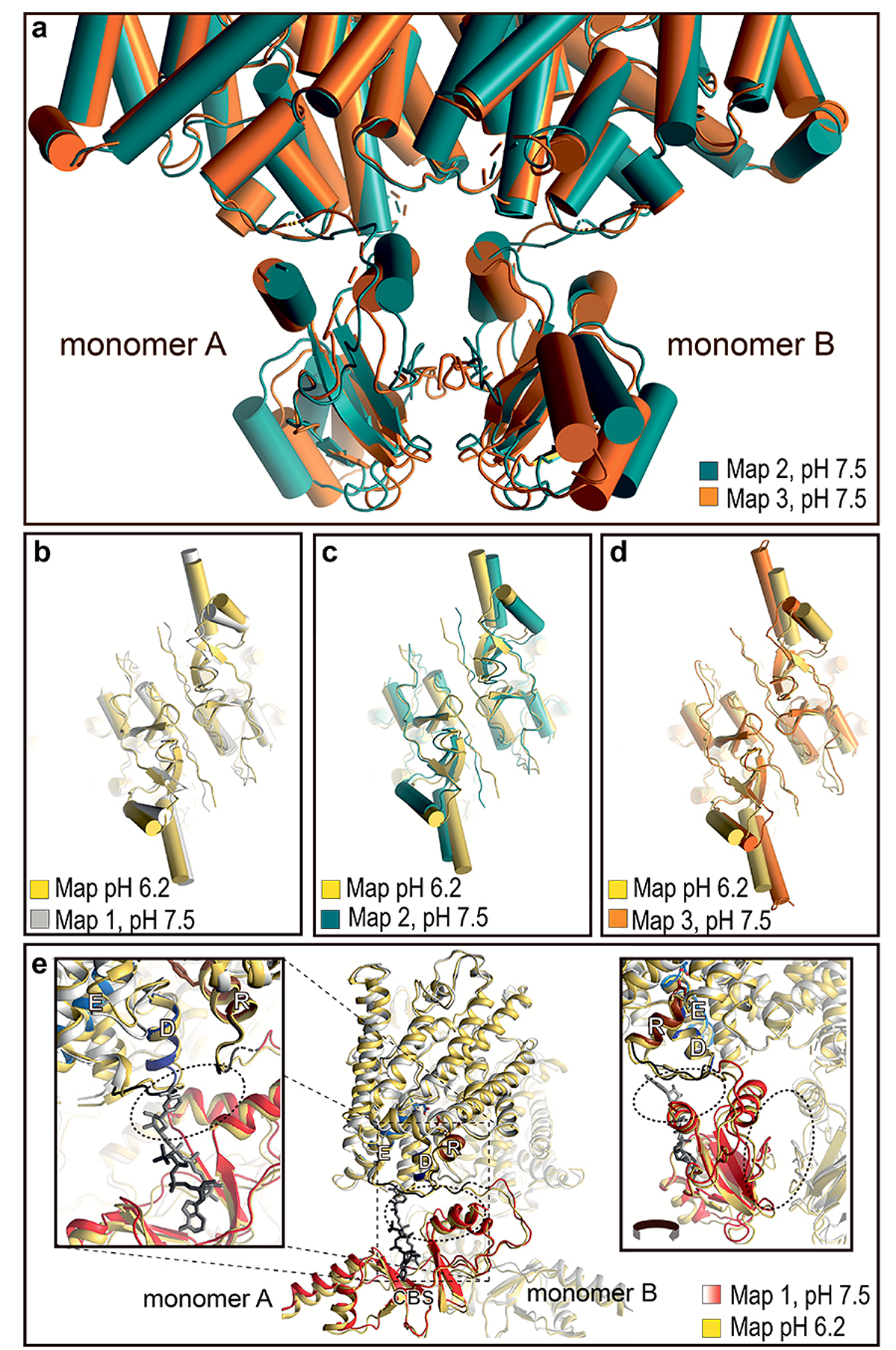

Supplement: S8 Fig — Alternative views of Fig 3 (colored identically). (a) Alternative view of Fig 3B. (b–d) Identical view as in panel a with comparisons of the structures derived from Maps 1–3 (pH 7.5) and the pH 6.2 map (aligned as in Fig 3A), respectively. (e) Identical view as Fig 3C, including the structure determined at pH 6.2. Helices are labeled with white letters throughout. CBS, cystathionine-β-synthase. (TIF) [file pbio.3000218.s008.tif]

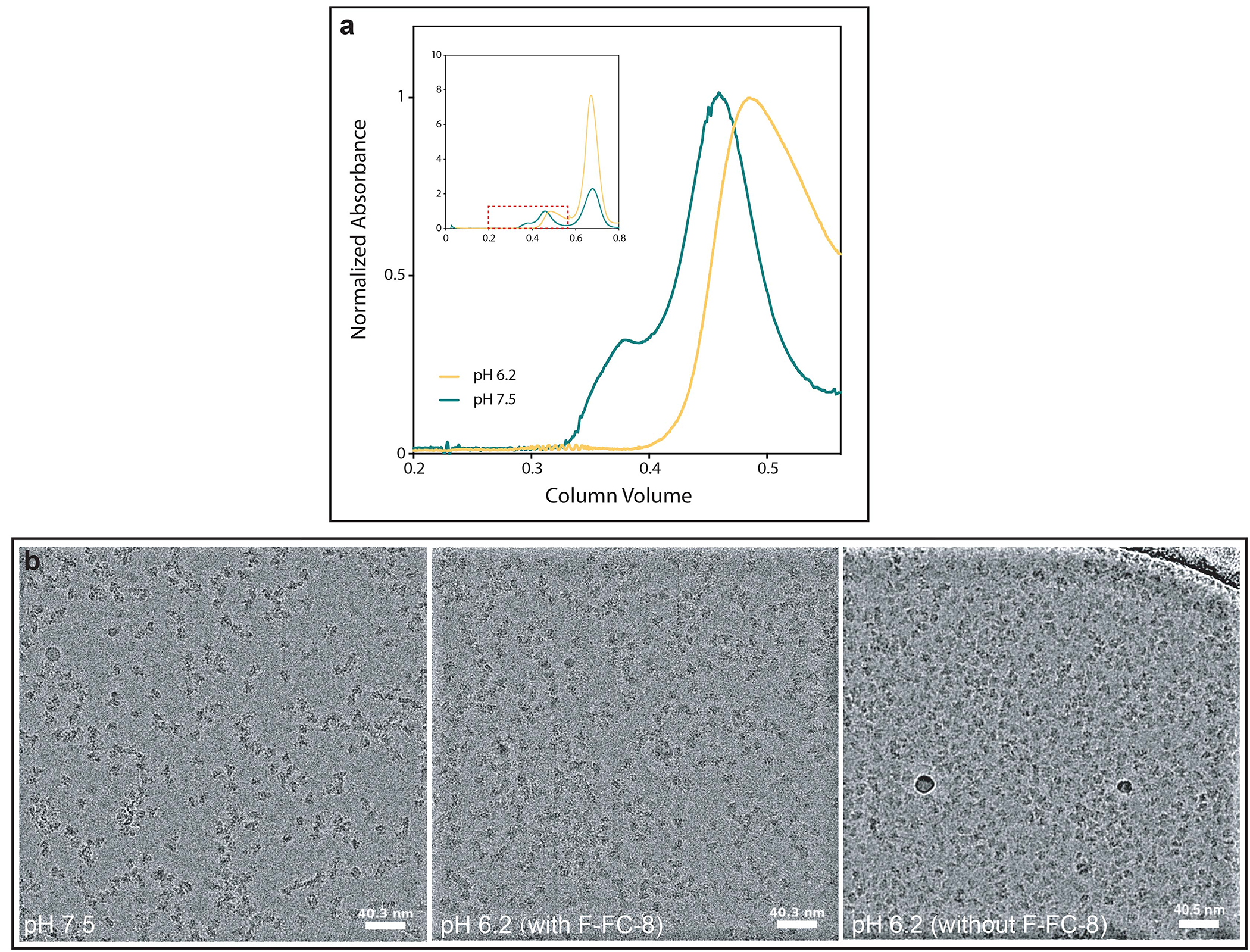

Supplement: S9 Fig — (a) Size-exclusion chromatography profiles of ClC-1 at pH 7.5 and 6.2. The protein peak at pH 6.2 is shifted toward a higher retention volume indicating a more compact ClC-1. The peak appearing at 0.7 CV represents the signal from free PMAL-C8 amphipol. (b) Micrographs for the pH 7.5 (left) and 6.2 (middle and right) data sets (with and without F-FC-8, respectively), indicating worse behavior of the pH 6.2 sample. CV, Column volume; F-FC-8, fluorinated fos-choline-8. (TIF) [file pbio.3000218.s009.tif]

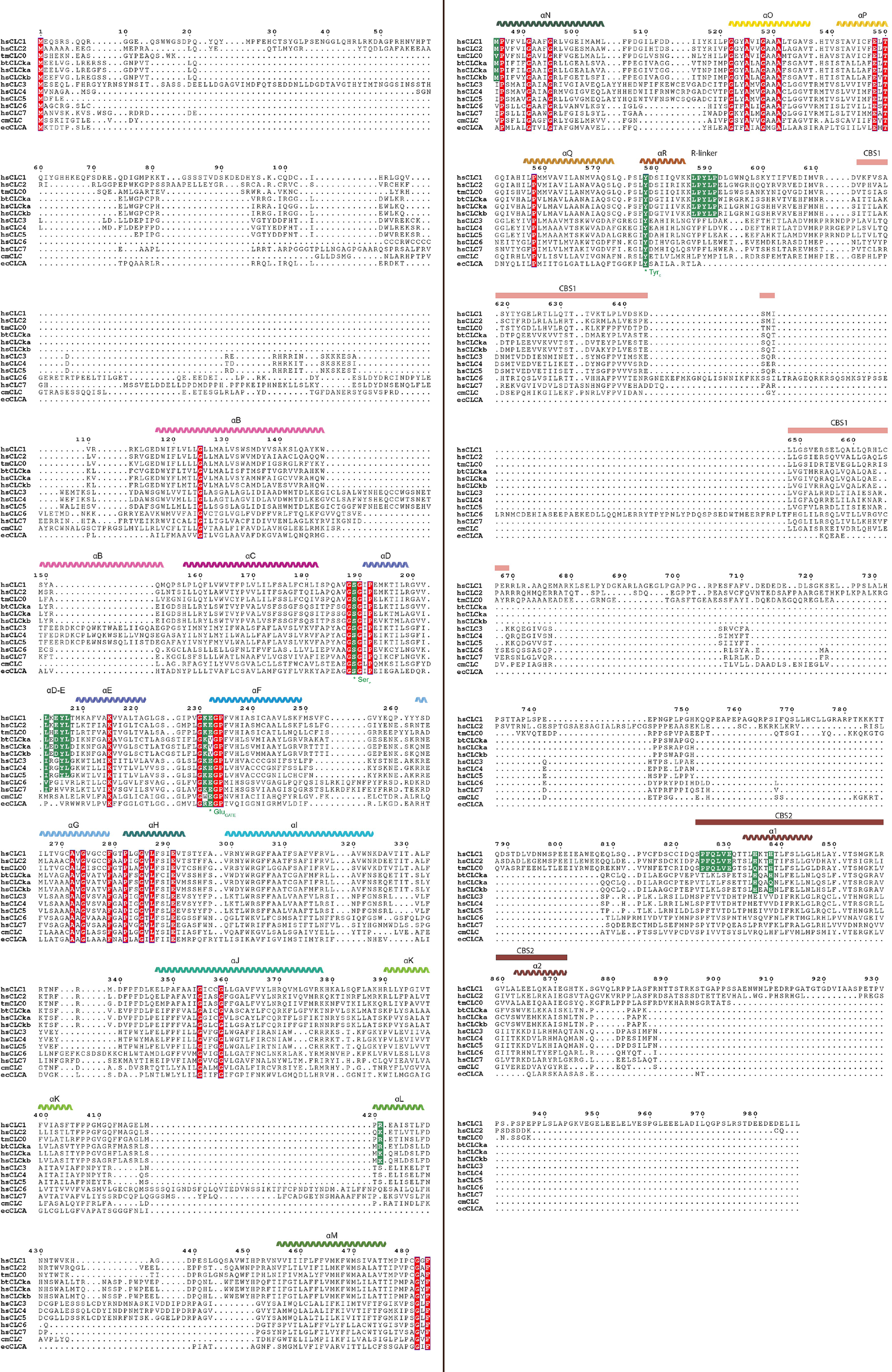

Supplement: S10 Fig — All human CLC members and structurally determined CLC proteins are displayed. Secondary structure elements are pinpointed, and conserved residues are highlighted in red and green, the latter representing residues relevant for the function of ClC-1 discussed in this work. CLC, chloride channel. (TIF) [file pbio.3000218.s010.tif]

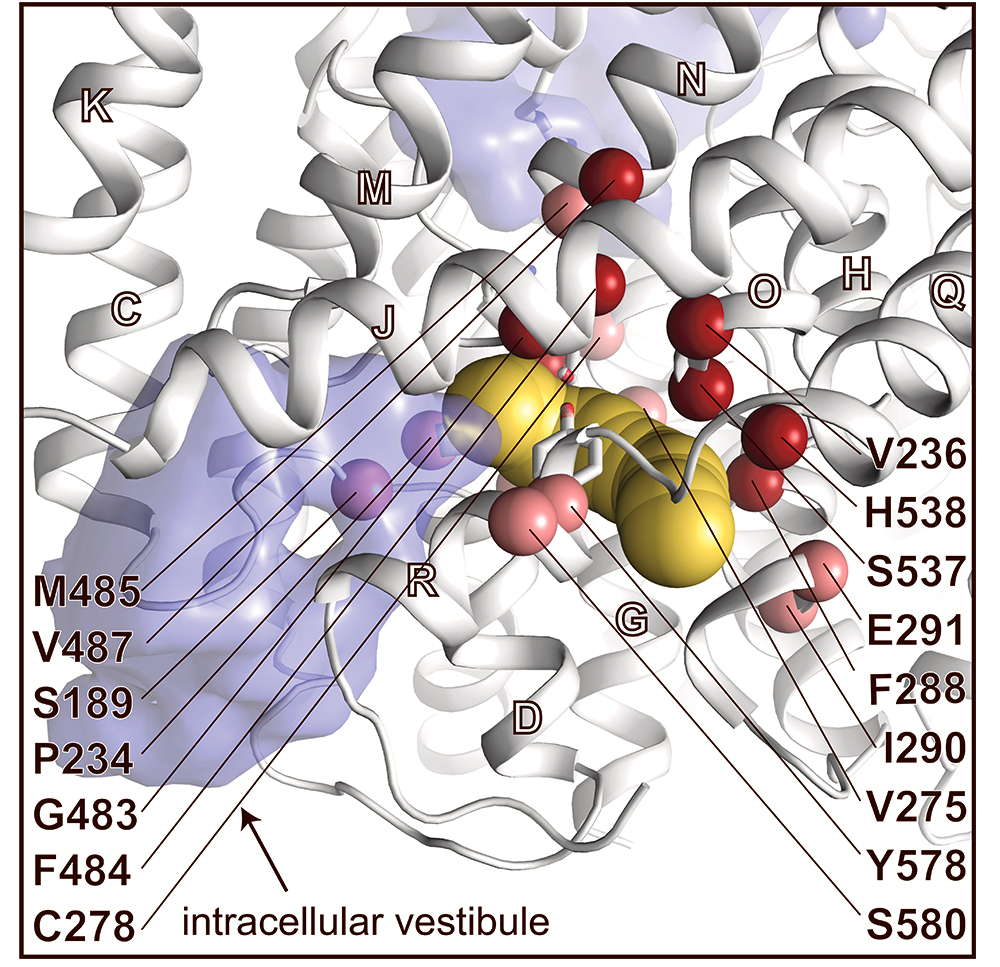

Supplement: S11 Fig — Alternative views of Fig 4E and 4F (colored identically). 9-AC, 9-anthracene-carboxylic acid. (TIF) [file pbio.3000218.s011.tif]
